# The role of socio-demographic and psychological factors in shaping individual carbon footprints in Finland

**DOI:** 10.1038/s41598-024-75302-7

**Published:** 2024-11-14

**Authors:** Elisa Sahari, Marja Salo, Nils Sandman

**Affiliations:** 1 Department of psychology and speech-language pathology, University of Turku, Turku, Finland; 2 Climate solutions unit, Finnish Environment Institute, Helsinki, Finland

**Keywords:** Carbon footprint, Climate change, Attitudes, Beliefs, Behaviour, Behavioural change, Human behaviour, Climate-change mitigation, Psychology and behaviour

## Abstract

Household consumption emissions are a major contributor to global greenhouse gas emissions, making them a vital target for emission reductions. While previous research has studied socio-demographic and situational factors in explaining the variation in individuals’ carbon footprints, a more comprehensive exploration of individual drivers, would benefit the development of effective and equitable mitigation policies. The current study examines associations between psychological and socio-demographic factors and carbon footprints of Finnish adults (*n* = 3,519). Income was found to be the strongest factor explaining the variation in carbon footprints. While environmental attitudes, perceived easiness and perceived importance of climate actions statistically explained differences in carbon footprints, the effect sizes were modest. The factors explaining variation in the carbon footprint varied across different domains (housing, travel, diet, and other consumption of goods and services), with psychological factors having a more substantial effect on diet compared to other domains. Overall, the findings suggest that tailoring policy interventions to reduce emissions from different domains to specific groups and factors could be beneficial. Furthermore, the greatest emission reduction potential lies among higher-income individuals. In the dietary domain, attitude-changing interventions could be more effective, while financial or choice architecture interventions might be better suited for other domains.

**Supplementary Information:**

The online version contains supplementary material available at 10.1038/s41598-024-75302-7.

## Introduction

Households play a vital role in climate change mitigation as their emissions account up to 72% of global^1,2^ and 61% of Finland’s^3^ consumption-based greenhouse gas (GHG) emissions. Consumption-based emissions, also called carbon footprints, refer to direct and indirect GHG-emissions of utilities, goods and services consumed by a nation, household or an individual^4^. In the current study, we focus on individual carbon footprint tied to out-of-pocket expenditure—namely, housing, travel, diet and consumption of other goods and services. We include emissions from the use and production of these goods and services, but exclude emissions from use of public services such as education and healthcare, following the precedent set by previous studies^3,5^. This exclusion was made as emissions from public services are shared resources and thus, cannot be directly controlled by individual choices. The aim of the study is to analyse carbon footprint components to clarify how individual characteristics affect the emissions, providing information for designing effective and targeted mitigation strategies.

In countries with high average income, such as Finland, individual carbon footprints are unsustainably high. In Finland the average individual carbon footprint has varied between 7 and 10 tonnes of CO_2_e during 2012–2021^3^. In order to achieve the targets outlined in the Paris Agreement, it would be necessary to lower the global average carbon footprint to 2.5 t CO_2_e per person by 2030 and to a mere 0.7 t CO_2_e by 2050^6^. Thus, there is a need for policy measures to reduce individual climate impact. While the practise of calculating individual carbon footprints have rightfully been criticized for, among other things, displacing the responsibility for emission reductions to individuals^7^, it can be utilized as an productive tool. For example, carbon footprints can serve as a quantifiable indicator for the necessity of household emission reductions. Furthermore, by identifying specific sources of emissions, they can guide focused strategies for overall emission reduction.

Efforts to reduce emissions have focused on technical solutions and improving energy efficiency of production, with the goal of diminishing the carbon footprint of different consumption goods^8^. However, there is a growing acknowledgement of the potential of demand-side solutions to mitigate climate change^9^. These solutions include e.g. adopting low-emission modes of travel and increasing the consumption of plant-based proteins. The solutions vary in their mitigation potential. For instance, living car-free can reduce individual emissions by 3.6–0.6 t CO_2_e and shifting to a more plant-based diet by 2.1–0.4 t CO_2_e^10^. In contrast, recycling or using recycled materials, while important for other reasons, have only a marginal impact on individual GHG emissions^10^. Overall, depending on the specific area of application, demand-side solutions could reduce global emissions by 40–80%^11^. To create behavioural change that leads to significant emission reductions at national and global level, it is necessary to understand the factors that influence an individual’s lifestyle and consumption patterns and thus the size of their carbon footprint.

Pro-environmental behaviour, i.e. actions that aim to reduce an individual´s negative environment impact^12^, has been the focus of numerous studies. These studies have identified several factors influencing engagement in pro-environmental behaviours, including knowledge, values, attitudes, norms, perceived behavioural control, emotions such as climate concern, awareness of the consequences of actions and outcome efficacy, feelings of responsibility, and self-identity [e.g.^13,14–19^]. Additionally, political and worldviews^15^, psychological distance^20^, experience with climate change^21^, cognitive biases^15^, sociodemographic factors such as age and gender^15,22^ and contextual factors^15,18,22^ have been found to influence engagement in pro-environmental behaviour. Furthermore, several behavioural theories, including, theory of planned behaviour^23^, norm activation model^24^, and values-beliefs-norms theory^25,26^ have been applied to explain the engagement in pro-environmental behaviour.

When examining behaviours that aim to mitigate individual carbon footprint domains specifically, similar determinants have been identified. For example, sustainable travel choices seem to be influenced by perceived behavioural control, knowledge, and personal norms^27^, dietary choices by perceived behavioural control, environmental attitudes, and norms^28^ and heating choices by knowledge and environmental attitudes^29^. However, each domain is also shaped by its own contextual variables.

In addition to these more established factors, other individual-related factors, such as authoritarianism^30^, system justification^31,32^ and left-right political positioning^33^ are potentially connected to pro-environmental behaviour, but are less studied. Authoritarianism, encompassing the preference to submit to authority, to conform to traditional norms, and to punish those who deviate from these norms^34^, may influence pro-environmental behaviour through norms. As individuals with authoritarian tendencies are more likely to adhere to prevailing norms, they may engage less in environmental actions, if the norms are unsupportive^30^. Furthermore, authoritarianism has been found to be associated with anti-environmental attitudes^35^. System justification, refers to the preference for maintaining the status quo^36^. This tendency is often highlighted among individuals of higher social status, who benefit from the existing systems^37,38^. System justification can prove to be a challenge for climate change mitigation practices through resistance to change and holding on to the existing, high emission lifestyles^32^. Furthermore, individuals on the political right are generally more supportive of the capitalist economic system^39^ and less concerned about issues associated with it, such as climate change, compared to those on the political left^40^. This ideological difference can influence individual engagement in pro-environmental behaviours.

While the previous studies have identified associations between various factors and pro-environmental behaviour, these factors and actions might not lead to a low-emission lifestyle. People can engage in climate actions while the impact of these actions on their overall emissions might remain limited^41,42^. This could be due to, for example, the fact that individuals often underestimate the impact of significant actions like dietary changes, while overestimating the effectiveness of smaller actions such as recycling^43–45^. Moreover, individuals tend to overestimate their personal contribution, fostering a misconception about the actual impact of their climate actions^46^. Additionally, some studies have indicated a negative spill over effect^47^, wherein environmentally friendly actions in one aspect of life may be used to justify more emissions-intensive choices in other areas. Due to these reasons, examining single or even multiple pro-environmental actions might not be a reliable indicator of actual low emission lifestyle. To circumvent this limitation, the current study aims to investigate factors affecting individuals carbon footprint as it provides a more robust measurement of emissions linked to an individual’s lifestyle than engagement in pro-environmental actions.

Previous research that has examined the influence of socio-demographic, geographical, and technological factors on the carbon footprints of individuals and households [i.e. ^48–50^] have identified that income, in particular, significantly influences carbon footprints^42,51–61^. Higher income enables individuals to consume more and engage in emission-intensive activities. In contrast, the impact of psychological factors, such as attitudes and beliefs, on the carbon footprint has less clear association: Environmental attitudes and social norms, together with income, has been connected to individual carbon footprint, albeit with small effect size^62^. Similar results have been reported between environmental concern and carbon footprint^63^, pro-environmental values and carbon footprint^5^ and environmental self-identity and carbon footprint^57^.

The objective of the current study was to explore associations of sociodemographic and psychological factors with individual carbon footprints in Finland, in order to identify possible targets for interventions aimed at reducing emissions related to individuals´ consumption choices. Our aim was to determine how various sociodemographic and psychological factors are associated with overall carbon footprint as well as the carbon footprint of housing, travel, diet and consumption of goods and services. We aspired to find out if the previously recognised findings replicate in the context of Finland, while also studying for less established factors. While we anticipated replicating previously identified associations in this study, our approach was more exploratory than hypothesis-testing. Our goal was to investigate how various factors influence the carbon footprint within the context of a Nordic country and determine the relative importance of these factors when considered together.

The psychological factors we explored included climate concern, knowledge on climate change, knowledge on climate actions, perceived behavioural control as easiness of climate actions, environmental attitudes, preceived engagement in pro-environmental behaviour, and the belief whether climate actions matter, referring to the awareness of the consequences of actions or outcome-efficacy^64^, and perceived experience of climate change. Based on previous research, we expected lower levels of climate concern^15,22^, limited knowledge about climate change and climate actions^65^, reduced perceived behavioural control^15,18,22^, more negative environmental attitudes^15,18,22^, less perceived engagement in pro-environmental behaviour^46^, the belief that individual or collective climate actions do not matter^19^ and less perceived experience with climate change^21^ would result in greater carbon footprint.

Furthermore, we explored associations between less studied but potentially significant constructs of authoritarianism, system justification, and left-right political positioning. These factors were selected as understanding them is important for possible policy level interventions. For instance, for individuals with a tendency towards authoritarianism, interventions emphasizing social norms or authority-based approaches might be more effective^30^. Based on previous research, we predicted that tendency for authoritarianism^30^, higher system justification^66^, and right-wing political positioning^33^ would be associated with greater carbon footprints. Moreover, while we focused in this study on psychological factors, we recognized the significance of sociodemographic factors as highlighted in the previous studies [i.e.^48–50^]. Particularly income level was expected to have impact on carbon footprint, higher income being associated to greater carbon footprint.

## Materials and methods

### Data collection

The study utilizes data from CLIMATE NUDGE survey, which was designed and implemented as part of the CLIMATE NUDGE consortium at the University of Turku. The survey explores various psychosocial aspects related to climate change. The survey was collected by Kantar TNS from 22 April 2022 until 16 May 2022. A sample of 3600 participants was recruited from a panel of over 50 000 people that is maintained by Kantar. The panel is considered to represent Finnish general adult population in regards to age, gender, and place of residence. However, the sample that was collected has higher average age than the whole Finnish population. This could have been corrected by weighting the sample but since this approach would have introduced different sort of bias to the results, we chose to use the data as it is.

To avoid item order effects, the order of questions in the survey was partly randomized. The survey system did not let participants skip questions, therefore only fully completed questionnaires are included in the final sample. More information about the survey and data collection procedures can be found at: https://osf.io/3s8uc.

### Ethics statement

Respondents invited to participate in the CLIMATE NUDGE survey provided written informed consent via an online system before accessing the survey. They were informed of their right to halt survey completion at any stage, ensuring their responses would be deleted and not included in the study. The CLIMATE NUDGE survey study obtained ethical approval from the University of Turku’s Humanities and Social Sciences Ethical Board and all study procedures were conducted according to principles of Declaration of Helsinki.

### Participants

The survey was completed by 3 600 participants (*n*_female_= 2008, *n*_male_= 1587, *n*_other_ = 5), aged 19–90 years (M: 53.70, SD: 16.89). 3 528 of the participant’s completed the survey in Finnish, 62 in Swedish and 10 in English.

Exclusion criteria to the current study included having no permanent apartment (*n* = 4) or a postal code that did not correspond to any postal code in Finland (*n* = 18) as the estimation of the carbon footprint was not possible for these individuals. Furthermore, individuals having carbon footprints that deviated three or more standard deviations from the mean (*n* = 54) were excluded from the analyses, in order to filter out unrealistic responses. Additionally, participants who responded ‘other’ to the gender question were excluded from the analysis due to insufficient sample size (*n* = 5) to conduct analyses^67^. Therefore, the total size of the sample used in the analyses was 3 519.

### Measures

The study utilized the following survey items: respondent’s household income, gender, age, level of education, concern related to climate change, self-perception of how often one considers the environmental impact of their actions, how easy the respondent finds climate actions, how important the respondent perceives individual climate actions, how important the respondent perceives climate actions in Finland, perceived knowledge about climate change and perceived knowledge about climate actions. If the respondent was not able estimate their household income (*n* = 202), their participation in climate actions (*n* = 67), their knowledge about climate actions (*n* = 106), or importance of individual actions (*n* = 64) or climate actions in Finland (*n* = 51), the variables were considered as missing values. Furthermore, if the respondent reported that they do not consider the environmental impact of their actions and reported not being interested in the environmental impact of their own lifestyle, the item considering how easy the respondent finds climate actions was considered as missing value (*n* = 182). It should be noted that the treatment of missing values poses limitations to the interpretation of the models, as participants are excluded from the analysis in a non-random manner, particularly where the variable of easiness of climate action is considered. Consequently, the models presented in the main text do not include individuals who do not consider their environmental impact at all. This should be considered when interpreting the results.

Urbanicity of the respondent’s residential area was determined by respondents’ postal code and urban-rural classification meter created by the Finnish Environment Institute, Syke^68^. Based on the classification meter, we created a dichotomous urbanicity variable (*non-urban*,* urban*). A simple dichotomous variable was chosen, as the main differences in the carbon footprint related to the urbanicity of the respondent’s residential area were thought to depend on whether the respondent lived in or outside of a city. For example, residents in rural areas typically have to larger living spaces^69^ but have limited public transportation options^70^. Additionally, this classification simplified our statistical models, making them easier to interpret and helping to avoid issues like multicollinearity. Based on the urban-rural classification meter, sparsely populated rural areas, core rural areas, rural centres, peri-urban rural areas, urban peripheries were classified as non-urban and outer urban and inner urban areas were classified as urban.

Left-right wing political positioning was determined based on which party the respondent would vote for if there was an election. The respondents were divided into three groups: right-wing, left-wing and other based on a survey commissioned by the Finnish Ministry of Justice^71^.

Respondents’ attitudes towards the natural environment were measured by a shortened Finnish and Swedish translated version of the Environmental Attitudes Inventory (EAI-12)^72^. The EAI measures respondent’s views about the importance of the environment and beliefs on whether natural environment should be protected or whether humans have the right to alter it as they see fit. Our shortened 12-item EAI version included one item from each of the 12 EAI-scales (see table S1.2). A unidimensional environmental attitude variable was created from the 12 items. A higher score indicated a more positive attitude towards the natural environment. Distribution of the EAI-12 variable is presented in figure S1.1.

System justification and support for the status quo was assessed by a version of Kay and Jost’s System Justification Scale (SJS)^73^. The scale consists of eight statements on respondents’ perceptions of the fairness, legitimacy, and justification of the prevailing social system (see table S1.4). The original eight statements were translated and modified to fit Finnish context. A higher score in unidimensional SJS construct represented a higher support on the prevailing social system. Distribution of the SJS variable is presented in figure S1.1.

Respondent’s tendency to favour authoritarian policies was measured with a modified version of the Very Short Authoritarianism Scale (VSA-3)^74^ (see table S1.3). A higher score on a unidimensional VSA-3 construct indicated greater tendency for conservatism and favouring authoritarian policies. Distribution of the VSA-3 variable is presented in figure S1.1.

The experience of climate change variable was defined on the basis of three questions asking whether the respondent had noticed changes due to climate change or whether climate change had affected their life or the lives of people they know.

More detailed information on the measures can be found in Supplementary material S1. Table S1.1 provides an overview of the distributions and respondent counts for each item.

### Carbon footprint estimation

The carbon footprint of the participants was estimated based on their answer to 51 questionnaire items related to housing, travel, diet and consumption of goods and services. A consumption-based approach was adopted [see e.g.^75,76^] i.e., embodied greenhouse gas emissions are also included. This means, for instance, that car driving emissions include fuel production and car manufacturing emissions in addition to GHG-emissions of fuel combustion during driving. A detailed description of the carbon footprint estimation is presented in Supplementary material S2.

GHG intensities for energy use in housing, travel, diet, and consumption of other goods and services have been tailored for this specific dataset. Intensities for Finnish conditions, production and consumption have been used when necessary and available.

To estimate individual carbon footprint, we distinguished between individual choices (diet and expenditure on goods and services) and choices related to the consumption of the individual´s household (e.g., housing) (see table S2.1). To allocate footprints from shared resource use, we divided the total household emissions by OECD consumption units for the household^77^. The OECD consumption unit distributes emissions in a manner that results in most emissions being attributed to adult household members.

Carbon intensity estimation was guided by the purpose of this research: To estimate the carbon footprint of each respondent by considering key differences in consumption patterns and emission intensities to see if and how carbon footprints vary across respondents (see tables S2.2–S2.6). Data collected in the survey is extensive and enables estimation of carbon footprints so that results reflect differences in consumption patterns. However, it is not as comprehensive or detailed as estimations based on household surveys conducted by statistical offices [e.g.^51,53^]. Thus, average carbon footprints estimated in this study may differ from footprints presented in other studies.

### Analysis

To explore correlations between the used variables and to ensure that there was a linear relationship between the explanatory variables and the carbon footprint, pairwise Pearson correlations were calculated. Pearson correlations were also calculated for categorical variables, i.e. gender, urbanicity, and left-right political positioning, to explore their relationships with the other explanatory variables. Dichotomous variables (female/male, non-urban/urban, right/left-wing positioning) were used in the calculations. Although Pearson correlation is typically used for continuous variables, it can also provide useful insights into the strength and direction of relationships involving dichotomous variables^78^. To further study associations between carbon footprint per consumption unit and the categorical variables, one-way analysis of variance was utilized. Throughout the study, ordinal variables were treated as continuous, in order to maintain straightforward and interpretable models. This approach was justified as each displayed a significant linear relationship with the dependent variable.

To study how different factors, explain variation in the size of carbon footprint we constructed a hierarchal multiple linear regression model, using two blocks of independent variables. The first block of independent variables consisted of sociodemographic factors, i.e., gender, age, household income and urbanicity and the second block contained political positioning, climate concern, EAI-12, SJS and VSA-3. Total carbon footprint per consumption unit was used as dependent variable. These analyses were pre-registered in the Open Science Framework (https://osf.io/aky68).

In addition to the preregistered models, we built a model that included additional psychological variables. The variables were decided based on the correlations and the variables that had a significant correlation to the carbon footprint, were chosen. Additionally, if a variable had a strong (PCC > = 0.60) correlation with another variable, only the one with a stronger correlation with the total carbon footprint was chosen. The additional variables included: perceived easiness of climate actions; perceived importance of climate actions in Finland and perceived knowledge about climate change. Climate concern variable was removed from the model due to a high correlation with the EAI- 12 (PCC = 0.61, *p* < 0.001). Furthermore, an interaction term between environmental attitudes and income was included in the model, we presumed that environmental attitudes would exhibit a more significant impact on the carbon footprint within high-income households. This assumption stemmed from the notion that individuals with higher incomes typically possess greater resources to adopt environmentally friendly choices, such as investing in geothermal heat pumps or electric cars. The interaction term was constructed by subtracting the mean values of the household income and EAI-12 from each of the values and then multiplying them together.

Furthermore, we built regression models, similar to the models outlined above, with the carbon footprint of travel, housing, and other goods and services as dependent variables. Additionally, we constructed a logistic regression model to examine the impact of dietary carbon footprint. Due to the limited number of participants in the vegan and high-meat diet groups, we combined the dependent variable into three categories: vegan and pesco-vegetarian (*n* = 190), low meat (*n* = 507), and average to high meat consumption (*n* = 2822). The average to high-meat diet was used as reference group.

To streamline our analysis and mitigate the issue of sparse data, we aggregated age, education, household income, perceived climate knowledge, perceived easiness of climate actions into fewer categories (see Table 5). The rationale for dividing the sociodemographic variables into groups was based on their reflection of distinct life situations, ensuring practicality and interpretability. Age categories were designed to capture meaningful life stages, such as young adults and retirees. Similarly, the categorization of income groups aimed to represent various wage levels while maintaining clarity and relevance. Additionally, scores from the EAI-12, VSA-3, SJS were rounded to the nearest whole number, consistent with their measurement scales, in order to reduce the number of groups.

To control for multiple hypothesis testing, we used alpha level of 0.01 throughout the study. Eta squared, R^2^, ∆R^2^, regression coefficients and Odds Ratios were used as estimates of effect size were applicable. Analyses were carried out in IMP SPSS Version 28.0 and in Python 3.9.

During the preparation of this work the authors used ChatGPT in order to improve text comprehensibility and grammar. Furthermore, ChatGPT was used to help to write and enhance Python code segments. After using this tool, the authors reviewed and edited the content as needed and take full responsibility for the content of the publication.

## Results

### Relationships between the variables

**Fig. 1:**
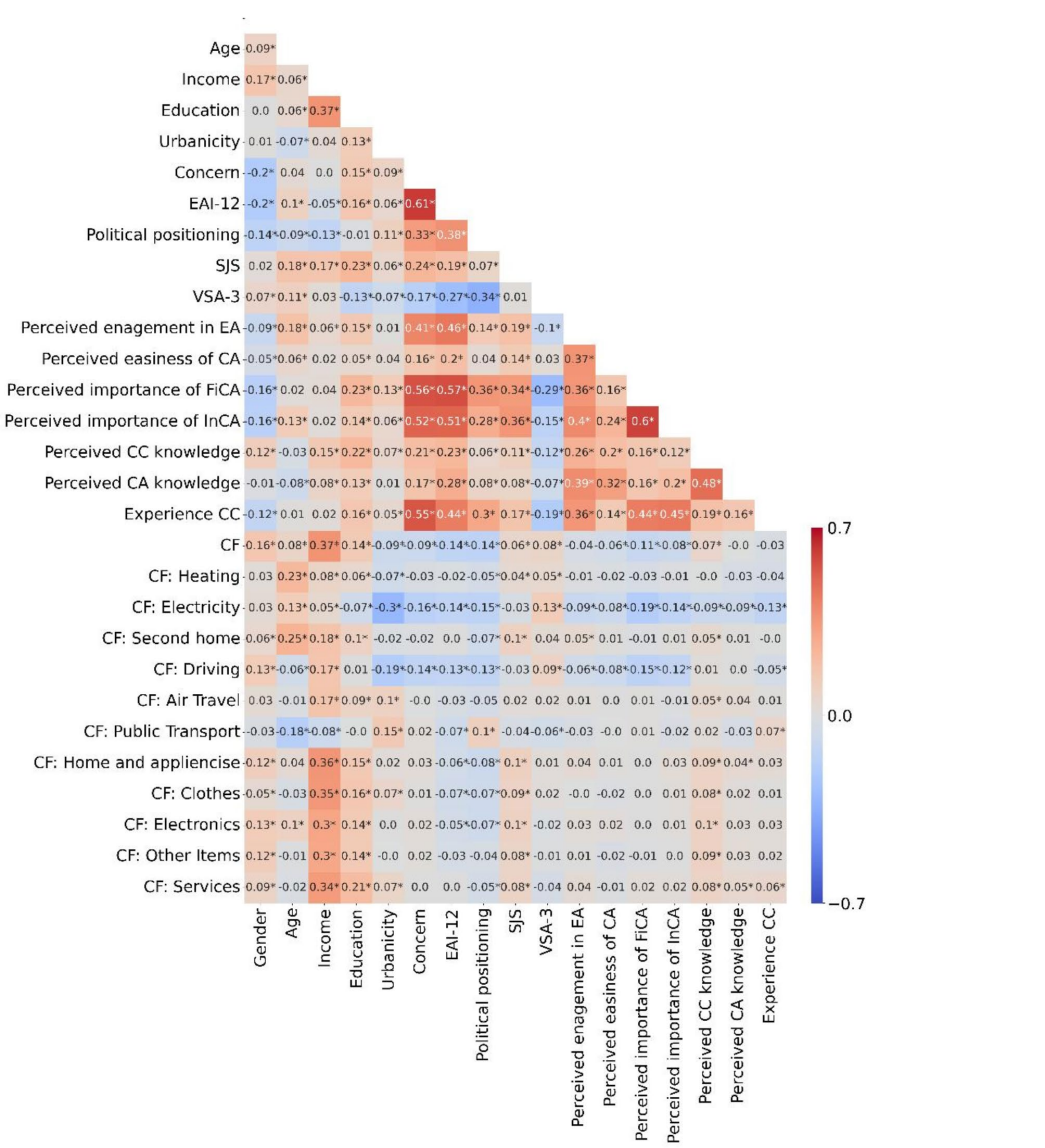
Pairwise Pearson correlation coefficients between the explanatory variables and the explanatory variables and carbon footprints. FinCA = perceived importance of climate action in Finland, InCA = perceived importance of climate action, CC = climate change, CA = climate action, * significant at p-value 0.01, female was used as the reference group for gender, right-wing for political positioning, and non-urban for urbanicity.

Pairwise correlations between the explanatory variables and carbon footprints were examined using pairwise Pearson correlations. The total carbon footprint showed a moderate correlation with income (PPC = 0.37, *p* < 0.001) and weak, but significant correlations with gender (PPC = 0.16, *p* < 0.001), age (PPC = 0.08, *p* < 0.001), education (PPC = 0.14, *p* < 0.001), urbanicity (PPC = -0.09, *p* < 0.001), concern (PPC = -0.09, *p* < 0.001), EAI-12 (PPC = -0.14, *p* < 0.001), political positioning (PPC = -0.14, *p* < 0.01), SJS (PPC = 0.06, *p* < 0.001), VSA-3 (PPC = 0.08, *p* < 0.001), perceived easiness of climate action (PPC = -0.06, *p* = 0.001), perceived importance of climate action in Finland (PPC = -0.11, *p* < 0.001), perceived importance of individual climate action (PPC = -0.08, *p* < 0.001), and perceived climate change knowledge (PPC = 0.07, *p* < 0.001). Furthermore, EAI-12 and climate concern (PPC = 0.61, *p* < 0.001), along with the perceived importance of climate action in Finland and individual climate action (PPC = 0.60, *p* < 0.001), were strongly correlated. The pairwise correlations are presented in Figure 1. The results of the ANOVA analyses, with categorical variables as predictors and carbon footprint as the dependent variable, are presented in tables S1.5–S1.7.

### Determinants of the total Carbon Footprint

**Fig. 2:**
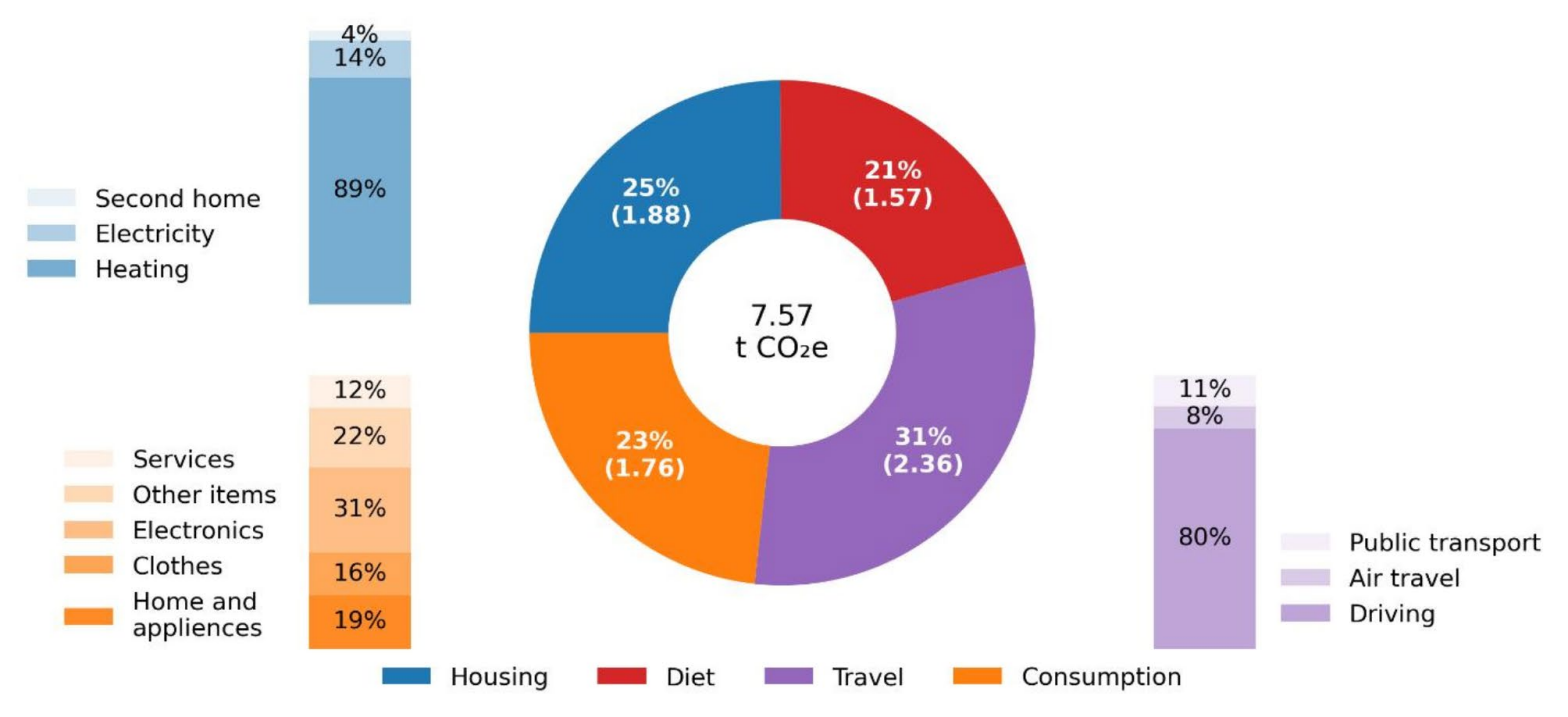
The average carbon footprint by carbon footprint domains. The average carbon footprint of the domain is shown in the brackets in t CO_2_e.

Average annual carbon footprint per consumption unit, estimated on the basis of survey items, was 7.57 t CO_2_e (SD: 3.06). The total carbon footprint consists of the sum of carbon footprints of housing, travel, diet and other consumption of goods and services. The average carbon footprints of the domains were 1.88 t CO_2_e (SD: 1.59) for housing, 2.36 t CO_2_e (SD: 1.78) for travel, 1.56 t CO2e (SD: 0.15) for diet and 1.76 t CO_2_e (SD: 1.38) for other consumption of goods and services. The average carbon footprint across the carbon footprint domains are presented in Fig. 2. Notably, the carbon footprint of diet was a categorical variable, with categories for vegan, vegetarian, low meat, average and high-meat diets. However, for the sake of simplicity, the average carbon footprint of the diet is utilized here. The distributions of the carbon footprint domains are presented in figure S1.2.

The impact of various factors on the total carbon footprint was analysed using multiple models. Initially, a simpler hierarchical multivariate linear model was employed, with the results detailed in supplementary material S1 table S1.8. The final, more comprehensive model included socio-demographic factors – gender, age, household income, and urbanicity of the respondent’s residential area – political positioning, Environmental Attitude Inventory (EAI-12)^72^, System Justification Scale (SJS)^73^, Very Short Authoritarianism Scale (VSA-3)^74^, perceived easiness of climate actions, perceived knowledge about climate change, perceived importance of climate actions in Finland, and the interaction between EAI-12 and household income as independent variables. The model explained variation in the size of the carbon footprint better than an empty model F(14, 3131) = 49.20, p = < 0.001, adjusted R^2^ = 0.18. Based on the model, individuals with older age, male gender, higher household income, who lived in non-urban areas, who had more negative environmental attitudes, perceived more difficulty in engaging in climate actions and considered climate actions in Finland less important had a greater carbon footprint but these associations had weak effect sizes. Strongest predictor of the carbon footprint in the model was household income (*p* < 0.001, ∆R^2^ = 0.09) with small effect size. The results are presented in Table 1.

**Table 1:** Results of multiple linear regression model with the total carbon footprint as the dependent variable.

	B	SE	Stand. B	[LL, LU]	*p*	∆*R*^2^	VIF
Intercept	7.262	0.553	**-**	[5.837, 8.687]	< 0.001*	**-**	**-**
Age	0.008	0.003	0.045	[0.000, 0.016]	0.008*	0.002	1.11
Gender: Women^1^	-0.392	0.106	− 0.063	[-0.665, -0.119]	< 0.001*	0.004	1.13
Household income	0.513	0.028	0.325	[0.439, 0.586]	< 0.001*	0.085**	1.24
Level of education	0.125	0.059	0.039	[-0.027, 0.278]	0.034	0.001	1.32
Urbanicity: Urban^2^	-0.603	0.109	− 0.091	[-0.885, -0.321]	< 0.001*	0.008	1.04
EAI-12	-0.273	0.076	− 0.077	[-0.468, -0.078]	< 0.001*	0.003	1.73
Politics: Left^3^	-0.125	0.124	− 0.019	[-0.445, 0.196]	0.316	< 0.001	1.44
Politics: Other^3^	-0.276	0.146	− 0.035	[-0.652, 0.099]	0.058	0.001	1.31
SJS	0.136	0.083	0.030	[-0.078, 0.349]	0.101	0.001	1.3
VSA-3	0.069	0.072	0.017	[-0.117, 0.255]	0.340	< 0.001	1.23
Perceived easiness of CA	-0.218	0.067	− 0.055	[-0.391, -0.046]	0.001*	0.003	1.1
Perceived CC knowledge	0.163	0.063	0.046	[0.000, 0.326]	0.010	0.002	1.21
Perceived importance of FiCA	-0.158	0.051	− 0.067	[-0.290, -0.027]	0.002*	0.003	1.77
Income × EAI-12	-0.032	0.029	− 0.018	[-0.107, 0.043]	0.272	< 0.001	1.01

1: compared to men. 2 compared non-urban, 3: compared to right-wing, CC = climate change, CA = climate action, FiCA = Climate actions in Finland, * significant at p-value < 0.01, ** variable with highest ∆R^2^, VIF = variance inflation factor.

**Fig. 3:**
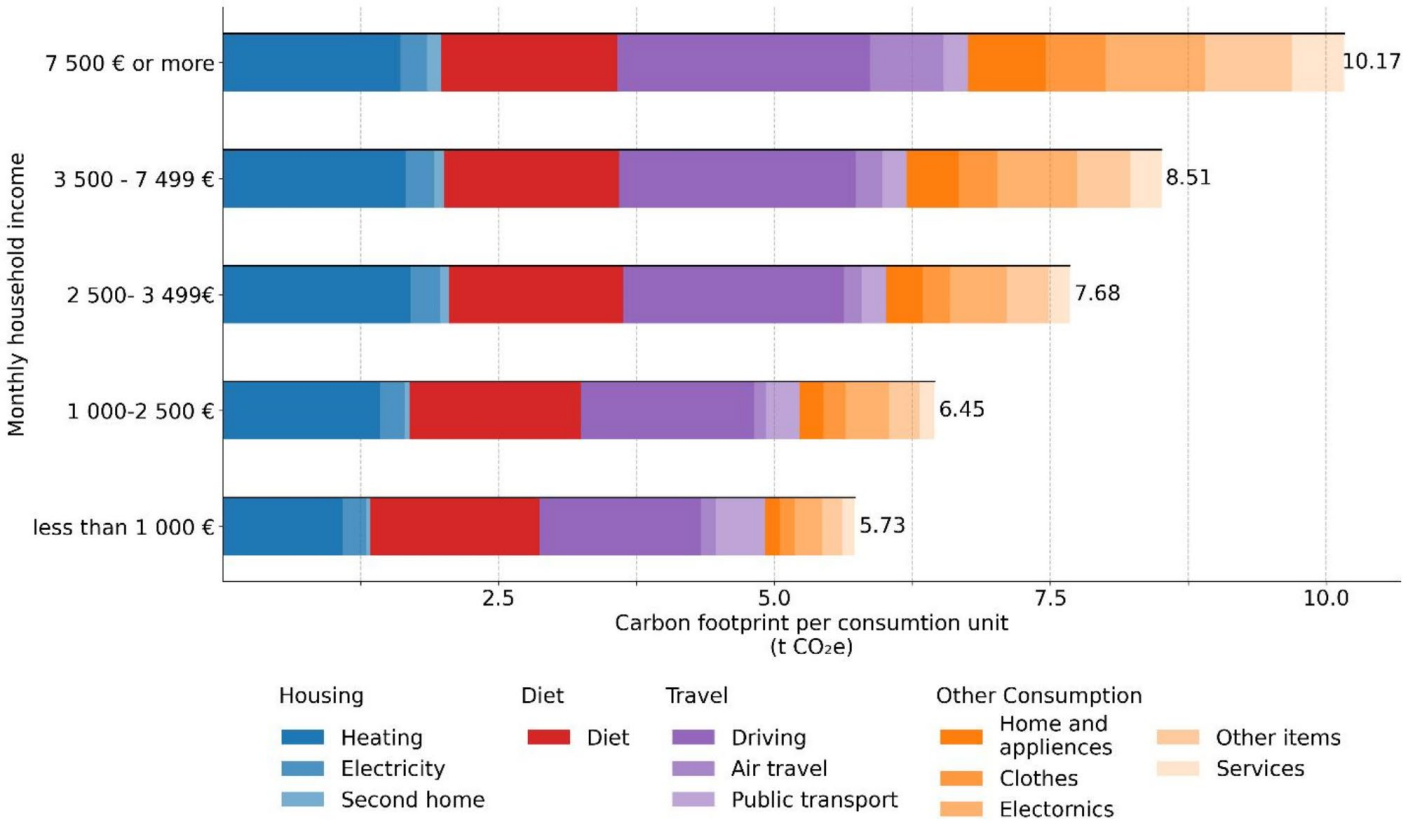
Average carbon footprint by consumption domain and household income.

Furthermore, as household income was the most prominent predictor of the total carbon footprint, we wanted to further illustrate its role as determining factor across the carbon footprint domains. Therefore, we divided participants into five income groups based on their household income: monthly household income less than 1 000 € (*n* = 231), 1 000–2 499 € (*n* = 1055), 2 500–3 499 € (*n* = 707), 3 500–7 499 € (*n* = 1421), 7 500 € or more (*n* = 157) and visualized the average carbon footprint per consumption unit across the carbon footprint domains. The visualization is presented in Fig. 3.

### Determinants of the Carbon Footprint domains

Linear regression models were conducted to analyse the determinants of the carbon footprint of housing, travel and other consumption of goods and services, reflecting the approach taken for the total carbon footprint. The results from the initial models are presented in supplementary material S1, tables S1.9–S1.11. The final model explained the variation in the size of the carbon footprint of housing better than an empty model F(14, 3131) = 20.05, p = < 0.001, adjusted R^2^ = 0.09. The results are presented in Table 2. Based on the model individuals with older age, higher household income and those who live in non-urban areas compared to urban areas had a greater carbon footprint of housing. Strongest predictor in the model was age (*p* < 0.001, ∆R^2^ = 0.05) with small effect size.

**Table 2:** Results of the hierarchical linear regression model with carbon footprint of housing as dependent variable.

	B	SE	Stand. B	[LL, LU]	*p*	∆*R*^2^
Intercept	1.060	0.303	**-**	[0.279, 1.842]	< 0.001*	**-**
Age	0.022	0.002	0.238	[0.018, 0.027]	< 0.001*	0.051**
Gender: Women^1^	0.038	0.058	0.012	[-0.112, 0.187]	0.517	< 0.001
Household income	0.058	0.016	0.071	[0.018, 0.098]	< 0.001*	0.004
Level of education	0.060	0.032	0.036	[-0.024, 0.144]	0.065	0.001
Urbanicity: Urban^2^	-0.297	0.060	− 0.086	[-0.451, -0.142]	< 0.001*	0.007
EAI-12	-0.041	0.042	− 0.022	[-0.148, 0.066]	0.320	< 0.001
Politics: Left^3^	-0.012	0.068	− 0.004	[-0.188, 0.164]	0.861	< 0.001
Politics: Other^3^	0.038	0.080	0.009	[-0.168, 0.244]	0.636	< 0.001
SJS	0.029	0.045	0.012	[-0.088, 0.146]	0.525	< 0.001
VSA-3	0.047	0.040	0.023	[-0.055, 0.149]	0.232	< 0.001
Perceived easiness of CA	-0.071	0.037	− 0.035	[-0.166, 0.023]	0.052	0.001
Perceived CC knowledge	0.014	0.035	0.007	[-0.076, 0.103]	0.694	< 0.001
Perceived importance of FiCA	-0.050	0.028	− 0.04	[-0.122, 0.022]	0.076	0.001
Income × EAI-12	0.001	0.016	0.001	[-0.040, 0.042]	0.951	< 0.001

1: compared to men. 2 compared non-urban, 3: compared to right-wing, CC = climate change, CA = climate action, FiCA = Climate actions in Finland, * significant at p-value < 0.01, ** variable with highest ∆R^2^.

The final model explained the variation in the size of the carbon footprint of travel better than an empty model F(14,3131) = 21.13, p = < 0.001, adjusted R^2^ = 0.08. The results of the model are presented in Table 3. Based on the model individuals with younger age, male gender, higher household income, who lived in non-urban areas, and perceived climate actions in Finland less important had a greater carbon footprint of travel. In the model household income was the strongest predictor albeit with small effect size (*p* < 0.001, ∆R^2^ = 0.02).

**Table 3:** Results of multiple linear regression model with the carbon footprint of travel as the dependent variable.

	B	SE	Stand. B	[LL, LU]	*p*	∆*R*^2^
Intercept	3.927	0.339		[3.054, 4.800]	< 0.001*	
Age	-0.015	0.002	− 0.142	[-0.020, -0.010]	< 0.001*	0.018
Gender: Women^1^	-0.238	0.065	− 0.066	[-0.405, -0.071]	< 0.001*	0.004
Household income	0.155	0.017	0.169	[0.110, 0.200]	< 0.001*	0.023**
Level of education	0.015	0.036	0.008	[-0.079, 0.108]	0.683	< 0.001
Urbanicity: Urban^2^	-0.311	0.067	− 0.081	[-0.484, -0.139]	< 0.001*	0.006
EAI-12	-0.108	0.046	− 0.053	[-0.228, 0.011]	0.019	0.002
Politics: Left^3^	-0.037	0.076	− 0.01	[-0.233, 0.159]	0.627	< 0.001
Politics: Other^3^	-0.190	0.089	− 0.042	[-0.421, 0.040]	0.033	0.001
SJS	-0.025	0.051	− 0.01	[-0.156, 0.105]	0.619	< 0.001
VSA-3	0.087	0.044	0.037	[-0.027, 0.201]	0.050	0.001
Perceived easiness of CA	-0.105	0.041	− 0.046	[-0.210, 0.001]	0.011	0.002
Perceived CC knowledge	0.065	0.039	0.032	[-0.035, 0.165]	0.092	0.001
Perceived importance of FiCA	-0.088	0.031	− 0.064	[-0.168, -0.007]	0.005*	0.002
Income × EAI-12	-0.002	0.018	− 0.002	[-0.047, 0.044]	0.928	< 0.001

1: compared to men. 2 compared non-urban, 3: compared to right-wing, CC = climate change, CA = climate action, FiCA = Climate actions in Finland, * significant at p-value < 0.01, ** variable with highest ∆R^2^.

The final model explained the variation in the size of the carbon footprint of other consumption better than an empty model F(14,3131) = 67.03, p = < 0.001, adjusted R^2^ = 0.23. The results of the model are presented in Table 4. Based on the model, men, respondents with higher household income, more negative environmental attitudes, greater support for societal status quo and higher self-reported understanding of climate change had a greater carbon footprint of other consumption. In the model household income was the strongest predictor with a small effect size (*p* < 0.001, ∆R^2^ = 0.13).

**Table 4:** Results of multiple linear regression model with carbon footprint of consumption as dependent variable.

	B	SE	Stand. B	[LL, LU]	*p*	∆*R*^2^
Intercept	0.566	0.242		[-0.057, 1.190]	0.019	
Age	0.001	0.001	0.010	[-0.003, 0.004]	0.545	< 0.001
Gender: Women^1^	-0.161	0.046	− 0.058	[-0.281, -0.042]	< 0.001*	0.003
Household income	0.290	0.012	0.407	[0.258, 0.322]	< 0.001*	0.133**
Level of education	0.057	0.026	0.040	[-0.010, 0.124]	0.028	0.001
Urbanicity: Urban^2^	0.013	0.048	0.004	[-0.110, 0.136]	0.788	< 0.001
EAI-12	-0.094	0.033	− 0.059	[-0.179, -0.009]	0.004*	0.002
Politics: Left^3^	-0.065	0.054	− 0.022	[-0.205, 0.075]	0.234	< 0.001
Politics: Other^3^	-0.128	0.064	− 0.036	[-0.292, 0.037]	0.046	0.001
SJS	0.117	0.036	0.058	[0.024, 0.211]	0.001*	0.003
VSA-3	-0.080	0.032	− 0.044	[-0.161, 0.002]	0.012	0.002
Perceived easiness of CA	-0.032	0.029	− 0.018	[-0.107, 0.044]	0.280	< 0.001
Perceived CC knowledge	0.088	0.028	0.055	[0.017, 0.159]	0.002*	0.002
Perceived importance of FiCA	-0.013	0.022	− 0.012	[-0.070, 0.044]	0.557	< 0.001
Income × EAI-12	-0.032	0.013	− 0.04	[-0.065, 0.001]	0.012	0.002

1: compared to men. 2 compared non-urban, 3: compared to right-wing, CC = climate change, CA = climate action, FiCA = Climate actions in Finland, * significant at p-value < 0.01, ** variable with highest ∆R^2^.

The determinants of the carbon footprint of diet were explored using logistic regression models, similar to the linear regression models used in other analyses, albeit with reduced variable categories (see Table 5). Due to the limited number of participants in the vegan and high-meat diet groups, the dependent variable was consolidated into three categories: vegan and pesco-vegetarian (*n* = 190), low meat (*n* = 507), and average to high meat consumption (*n* = 2822). Average to high meat diet was used as the reference group. The results from the initial model is presented in Supplementary material S1, tables S1.12–S1.13. The final model demonstrated a significant improvement in fit compared to the intercept-only model (χ² = 502.01, *p* < 0.001, Nagelkerke R-squared = 0.21). The results are presented in Table 5. Based on the model, respondents under 30 years old, compared to those over 40, along with female respondents, whose household income was between 3500 and 7500 € compared to those with income less than 2 500 €, those with more positive environmental attitudes, less supportive of authoritarian policies, who find climate actions very easy, as opposed to not too difficult or more difficult were more likely to adopt a vegan or pesco-vegetarian diet rather than an average or high meat diet. Furthermore, respondents completely disagreeing with the statement that climate actions are pointless in Finland, compared to those who mostly disagreed, showed a higher propensity for the vegan or pesco-vegetarian diet. Additionally, female respondents, those whose household income was less than 2500 euros compared to those with income between 3500 and 7500 euros, respondents with higher education, respondents who had more positive environmental attitudes, who completely disagreed with the statement that climate actions in Finland are pointless compared to those who mostly agreed, were more likely to opt for a low meat diet over an average or high meat diet. Gender exhibited the strongest associations with diet. Women were more likely than men to report vegan and pesco-vegetarian diets (OR 3.21) or low meat diets (OR 1.29).

**Table 5:** Multinomial logistic regression model with carbon footprint of diet as depended variable. Reference category is high meat diet. Risk is presented as exp(B) odd ratios.

Variable		*n*	Vegan or pesco-vegetarian			Low meat		
			*p*	OR	99% CI	*p*	OR	99% CI
Age	≥ 65 years	1058	< 0.001*	0.32	[0.15, 0.67]	0.087	1.44	[0.83, 2.49]
	50–64 years	779	< 0.001*	0.27	[0.12, 0.61]	0.950	1.01	[0.57, 1.80]
	40–49 years	556	< 0.001*	0.34	[0.15, 0.78]	0.015	0.54	[0.29, 1.04]
	30–39 years	467	0.450	0.81	[0.40, 1.65]	0.290	0.77	[0.41, 1.45]
	≤ 29 years	246						
Gender	Women	1739	< 0.001*	3.21**	[1.75, 5.89]	0.027	1.29**	[0.96, 1.73]
	Men	1367						
Household	≥ 7 500 €	106	0.438	0.61	[0.12, 3.12]	0.012	0.41	[0.16, 1.03]
Income	3 500–7 499 €	1222	0.006*	0.5	[0.26, 0.96]	< 0.001*	0.54	[0.38, 0.76]
	2 500- 3 499 €	638	0.028	0.56	[0.29, 1.11]	< 0.001*	0.58	[0.40, 0.84]
	≤ 2 500 €	1140						
Education	2. level or lower	1484	0.195	0.77	[0.47, 1.29]	< 0.001*	0.61	[0.45, 0.83]
	3. level or higher	1622						
Urbanicity	Non-urban	936	0.083	0.66	[0.36, 1.22]	0.283	1.13	[0.84, 1.53]
	Urban	2170						
EAI-12	Increase by 1 point^1^		< 0.001*	1.93	[1.36, 2.73]	0.009*	1.23	[1.00, 1.51]
Politics	Left-wing	1166	0.053	1.58	[0.86, 2.91]	0.196	1.18	[0.85, 1.64]
	Other	516	0.534	1.22	[0.54, 2.78]	0.158	1.26	[0.83, 1.91]
	Right-wing	1424						
VSA-3	Increase by 1 point^1^		0.002*	0.69	[0.51, 0.94]	0.036	0.86	[0.72, 1.03]
SJS	Increase by 1 point^1^		0.108	0.8	[0.57, 1.14]	0.037	0.84	[0.68, 1.04]
Perceived easiness of	Very difficult or difficult	310	0.014	0.37	[0.13, 1.04]	0.027	0.53	[0.25, 1.11]
CA	Not difficult or easy	1495	0.006*	0.43	[0.19, 0.95]	0.164	0.73	[0.40, 1.31]
	Easy	1111	0.080	0.6	[0.28, 1.27]	0.939	0.98	[0.55, 1.75]
	Very easy	190						
Perceived CC	No knowledge	706	0.053	1.71	[0.84, 3.46]	0.583	0.92	[0.61, 1.38]
knowledge	Poor	1371	0.401	1.24	[0.65, 2.37]	0.148	0.83	[0.60, 1.16]
	Mediocre or better	1029						
There is no	Completely agree	176	0.107	0.19	[0.01, 2.72]	0.297	0.74	[0.35, 1.56]
point in FiCA	Mostly agree	455	0.348	0.7	[0.27, 1.86]	0.008*	0.58	[0.34, 0.98]
	Neither agree or disagree	530	0.040	0.44	[0.16, 1.23]	0.183	0.78	[0.49, 1.26]
	Mostly disagree	848	0.006*	0.5	[0.26, 0.96]	0.062	0.77	[0.53, 1.11]
	Completely disagree	1097						
Income X EAI-12		0.426	1.05	[0.91, 1.20]	0.207	1.05	[0.96, 1.14]	

CC = climate change, CA = climate action, FiCA = Climate actions in Finland, * significant at p-value < 0.01, ** variable with highest significant OR, ^1^ promotion to the next aggregated group.

## Discussion

In this study, our aim was to provide a description of factors that explain differences in the size of individual carbon footprints in Finland. Through an exploration of socio-demographic and psychological factors, we identified income as the most significant factor explaining the variation in individual carbon footprint sizes. Among the psychological factors, environmental attitudes, perceived easiness of climate actions and perceived importance of climate actions in Finland were found to explain the differences in individual carbon footprints, although with small effect sizes. Furthermore, the outcomes highlighted that the carbon footprint domains are explained by different factors, with diet being notably influenced by psychological factors compared to other domains.

Our results reinforce previous findings on the strong connection between individual carbon footprint and income^42,51–61^. While income is known to have a strong connection with the overall emissions, the current results highlight that the role of income varies by consumption domain: Income emerged as the most significant factor explaining variation in carbon footprints associated with travel and consumption. However, while it was important for housing and diet, age for housing, and gender for diet were found to have even stronger associations for carbon footprint. The strong connection between income and consumption emissions indicate that individuals in households with higher incomes (i.e. average incomes and higher) possess the greatest potential for emission reductions. Figure 3 illustrates how, particularly, emissions from air travel and other consumption, potentially non-essential parts of the individual’s life, increase with household income. Consequently, interventions focused on mitigating household consumption emissions should be directed towards households with higher incomes. This rationale is supported not only by the fact that higher-income households have greater emissions but also by the understanding that individuals with more deposable income tend to have more choices regarding their lifestyle and have better chances to embrace environmentally friendly options that require investments, such as geothermal heat pumps and electric cars. Furthermore, reducing the larger carbon footprint can be more feasible compared to decreasing a smaller carbon footprint, primarily tied to essential needs.

Whereas prior research has acknowledged the influence of environmental attitudes and self-efficacy—perceived easiness of engaging in climate actions—on shaping climate-friendly behaviours^15,18^, our results elucidate their impact on overall lifestyles and carbon footprints. Although the effect sizes were small, the factors appear to influence various life decisions, impacting not only the overall carbon footprint but also the footprints associated with diet, and other consumption. Additionally, individuals with positive environmental attitudes also exhibited concern about climate change, as indicated by the strong correlation between these variables (Fig. 1). Therefore, both heightened climate concern and positive environmental attitudes appear to be associated with a lower carbon footprint. However, the impact of environmental attitudes was more pronounced in our data. While these analyses do not establish causation between the variables and carbon footprint, they do suggest that enhancing environmental appreciation and improving the perceived ease of climate actions could lower individuals’ carbon footprints to some extent.

Furthermore, based on the results, individuals who thought that taking-action to mitigate climate change in Finland matters, tended to have smaller carbon footprints. Additionally, these individuals also valued individual climate actions (Fig. 1). These factors can be understood as reflecting the belief in the positive consequences of both individual and collective climate actions^64^. Thus, the results provide confirmation for the idea that individuals are more likely to engage (or not engage) in actions if they believe in the positive (or negative) consequences of the actions. Thus, our findings suggest that improving the clarity and visibility of the impacts associated with collective and individual changes could foster reductions in individual carbon footprints. However, based on our analyses, we cannot determine whether individuals with less emission intense lifestyles subsequently believe that climate actions have a meaningful impact, or whether their belief in the significance of such actions motivates them to behave in this way, as is suggested by the norm activation model^24^.

When looking at the carbon footprint domains separately, we found that different variables emerged as significant factors explaining the variation in different carbon footprint domains. The difference between the carbon footprint of diet and other domains was particularly evident. Variation in domains other than diet was mainly explained by socio-demographic factors, particularly income. Incorporating political and psychological factors into the models within these domains only marginally increased their explanatory power. However, in the domain of diet, environmental attitudes were found to be one of the strongest explanatory factors, suggesting that psychological factors have a more significant impact on the carbon footprint of diet than other domains.

The models explaining the carbon footprint of housing and travel exhibited relatively low explanatory powers, and the psychological variables contributed minimally. This could be interpreted as individuals having less influence over their emissions related to housing and travel or perceiving actions within these domains less frequently as climate actions, or both. For instance, the considerable carbon footprint of housing in countries like Finland, attributed to substantial energy consumption required for heating during cold winter^60^, would supports the idea of a lack of agency. Additionally, individual choices for significantly reducing energy consumption related to housing are often limited due to the high cost of energy-efficient renovations, the infrequency of changing residences, and the lack of options available to renters. Interestingly, the initial bilateral correlations (Fig. 3) suggest a pattern where emissions from electricity and driving seem to have stronger correlations with the psychological factors than other aspects within their domains. This supports the notion that these actions within the housing and travel domains could more accessible or more often considered as climate actions. Previous studies also indicate that heating related choices and, for example, reducing house size are seen as more difficult and less effective than switching to green energy^43^. However, the pattern and particularly its causality warrant further investigation.

Contrary to our hypotheses, authoritarianism, system justification, political positioning, and perceived climate change knowledge did not significantly explain the variation in the total carbon footprint when other factors were controlled. However, authoritarianism was associated with average or high meat diets rather than vegan, or pesco-vegetarian diets. This aligns with the notion that individuals with authoritarian tendencies adhere to norms and traditional values^34^. Furthermore, system justification was associated with higher carbon footprint of other consumption, possibly reflecting support for current consumption-oriented lifestyles^32^. The lack of significance in authoritarianism, system justification, and political positioning regarding the total carbon footprint could stem from their overlapping explanatory power, given their associations with similar beliefs and their connection to variables that more directly impacting emissions, such as perceived importance of climate actions. Additionally, higher perceived climate change knowledge was associated only to a higher carbon footprint in other consumption. This association may be due to the higher costs of sustainable goods, not factored into our carbon footprint estimation. However, this could also be due to the nature of the measure itself, which captures perceived rather than actual knowledge. This means that while individuals may perceive themselves as knowledgeable about climate change, some may hold misconceptions or incorrect beliefs. Furthermore, we assumed that positive environmental attitudes would have a more substantial influence on carbon footprint within high income households. However, the interaction between environmental attitudes and income did not emerge as significant factor in explaining the tola carbon footprint or any of its domains.

Furthermore, contrary to our hypotheses, engagement in environmental actions, perceived knowledge on climate actions, or experience with climate change did not significantly correlate with the carbon footprint. Interestingly, engagement in environmental actions and knowledge on climate actions showed a moderate correlation (Fig. 1.), suggesting that individuals who perceive themselves as taking climate actions also perceive themselves as having a good knowledge about them. The lack of significant correlation with carbon footprint suggests a bias between individuals’ perception and actually impactful climate actions. Thus, our data supports the previous idea that individuals may engage on actions, that do not necessary have significant emission reductions^79^ or they may overestimate their engagement in environmentally friendly behaviours^46^. This highlights the importance of accessible information on impactful actions while ensuring that these actions are accessible. However, the lack of connection could also be attributed to the possibility that individuals with lower incomes may not actively engage in climate actions, despite having smaller carbon footprints. This interpretation is supported by the weak positive correlation between household income and perceived engagement in environmental actions (Fig. 1). Thus, the relationship between perceived engagement and carbon footprint may not follow a linear pattern. Moreover, experience with climate change, did not correlate with carbon footprint. This aligns with studies suggesting that the issue of distance to climate change may be overrated^80^.

While we identified significant psychological factors that explained the variation in carbon footprints and its domains, it’s important to note that these associations were weak. This suggests that the current societal structures do not enable individuals to reduce their carbon footprint to a sustainable level. The fact that the respondents exhibited unsustainably high carbon footprints regardless of any of the measured variables supports this notion. Further highlighting this, even at the lowest income levels, essential needs (i.e. housing and diet) already exceed the target individual carbon footprint of 2.5 tCO_2_ by 2030 (see Fig. 3.)^6^. These limitations can arise from issues like inadequate infrastructure and technology or the influence of established social norms^81^, underscoring the necessity for structural and systemic changes, alongside behavioural adjustments, to achieve the targets.

Given the limited associations between the psychological variables and the size of carbon footprints, the study suggests that interventions focused on promoting single psychological factors, such as environmental attitudes, have only minimal impact on individuals’ carbon footprints, with the possible exception of diet. Instead of just targeting single psychological factors, interventions including financial incentives or choice architecture modifications targeting individuals with the greatest emission reduction potential, may be more effective. However, it’s imperative to ensure that transitioning toward less emission intense lifestyles is accessible and appealing to all individuals. This involves making sustainable choices easy, attractive, financially feasible, and readily accessible.

### Limitations

This study has limitations that need to be considered when evaluating the findings. The calculated individual carbon footprints are coarse estimates of actual emissions. Due to the constraints related to the survey, we had to use several averaged parameters to generate the individual estimates. Additionally, our data lack certainty regarding the distribution of resources among household members. Moreover, while analysing specific carbon footprint domains offers more detailed insights than focusing solely on the overall footprint, it’s crucial to recognize that factors within these domains, like driving and flying, may be influenced by different variables. This could potentially affect the conclusions drawn from this study. Furthermore, the reliance on self-reported behaviour introduces uncertainty to the results^82^.

There are also some limitations related to the explanatory variables. The EAI-12 measure was shortened from the original EAI by the authors and the measure has not been validated. Furthermore, when evaluating the ease of performing climate actions, we neglect to specify the particular actions under consideration. Individuals may perceive various actions as climate-related, with some being viewed as straightforward and others as more challenging. Moreover, while our study focuses primarily on climate change, we inquire about how often individuals consider the environmental friendliness of their actions. We recognize that environmental actions and climate actions are not entirely interchangeable, and we acknowledge this limitation in our data.

Finally, income was quantified based on household earnings, whereas carbon footprint was assessed on an individual basis. The household income does not account for household size, leading to a broad spectrum of households possessing similar income levels. For example, a single individual and a family of three, may report identical household incomes, yet the per capita disposable income varies significantly between them. Consequently, household income does not necessarily reflect the actual amount of money available for an individual to spend. However, relying solely on individual incomes would overlook the disposable income available to each person within a household, as household members earnings are often pooled together to cover expenses such as housing. We opted to focus on household income as it strikes a balance between reflecting the actual resources available to individuals within a household and maintaining interpretability of the model. Notably, changing the income variable to household income, personal income, or household income divided by consumption unit did not significantly alter the results.

## Conclusions

Our study aimed to understand the factors influencing individual carbon footprints in Finland. We found income to be the primary predictor, highlighting its significance across various carbon footprint domains. Environmental attitudes, ease of engaging in climate actions, and perceived importance of climate actions in Finland also played a role, though with small effect sizes. Moreover, our findings revealed that carbon footprint domains are influenced by distinct determinants, with psychological factors notably affecting dietary choices. These insights offer valuable guidance for tailoring interventions to reduce household consumption emissions and identify the groups most impacted by such measures. However, given the modest effects observed with psychological factors, interventions solely targeting single factors, like environmental attitudes, may have limited impact on individuals’ carbon footprints. Instead, focusing on multiple factors concurrently, and particularly households and climate actions with the greatest reduction potential, may be more effective.

While this study has provided valuable insights, several questions warrant further investigation. For example, the non-significant interaction between income level and environmental attitudes could be due to non-linear relationship between income, environmental attitudes, and carbon footprint. It is possible that environmental attitudes (or other psychological factors) are associated to smaller carbon footprints only within certain income groups, such as middle-income individuals. Understanding why some individuals maintain environmentally friendly lifestyles regardless of income, while others do not, could have important implications. To explore this further, alternative analytical methods beyond linear regression are needed, as well as an examination of factors within specific income brackets. Additionally, future research should consider factors not included in this study, such as social norms, which could have a substantial impact on behaviour.

## Electronic supplementary material

Below is the link to the electronic supplementary material.

Supplementary Material 1

## Data Availability

The current manuscript calculates carbon footprint of the participants. This involves utilizing 51 variables that are potentially indirect identifiers, such as postal code, size of the house, year the house was built, number and type of cars the participant owns etc. and these variables are contrasted with other indirect identifiers such as age, gender and education. While not direct identifiers, together these variables allow a relatively accurate determination of the participant’s place of residence which is information we are legally not allowed to share. Therefore, the full dataset is not publicly available. Anonymized version of the dataset is available upon request from the author for scientific purposes to a research institution in good standing via email from elisa.sahari@utu.fi or psychology@utu.fi. Details can be found at: https://doi.org/10.5281/zenodo.11369292.
