# Supplementary material for: The role of socio-demographic and psychological factors in shaping individual carbon footprints in Finland
Source: Sci Rep. 2024 Nov 14;14:27984. doi: 10.1038/s41598-024-75302-7 (PMC11564771; doi:10.1038/s41598-024-75302-7)
Supplement: Supplementary file 1 — Supplementary Material 1 [file 41598_2024_75302_MOESM1_ESM.pdf]

S1 Supplementary material

This supplementary material provides description of variables used in the study and their distributions. Statistical analyses not included in the main text are also presented.

Measures

Table S1.1 shows the explanatory variables used in the study and their distributional characteristics in the final sample.

Table S1.1. Explanatory variables, their response options and distribution of responses in final sample

Item	Response option		n	M	SD
Age				53.84	16.84
Gender	Female		1970		
	Male		1549		
Household income	Less than 1000 €		222		
	1000–1499 €		369		
	1500–2499 €		662		
	2500–3499 €		680		
	3500–4499 €		579		
	4500–5499 €		382		
	5500–6499 €		186		
	6500–7499 €		122		
	7500 € or more		115		
	I do not know		202		
Level of education	Primary education		262		
	Secondary education (Vocational school or High school)		1488		
	Lower third level degree		975		
	Higher third level degree		702		
	Doctoral degree or higher		92		
Urbanicity	Non-urban	Sparsely populated rural area	124		
		Core rural area	240		
		Rural centre	200		
		Peri-urban rural area	224		
		Urban periphery	305		
	Urban	Outer urban area	747		
		Inner urban area	1679		
Political positioning	Right-wing	National Coalition Party	711		
		Centre Party of Finland	305		
		The Finns	449		
		The Swedish People's Party of Finland	92		
		Christian Democrats	71		

	Left-wing	Greens	337		
		Social Democratic Party of Finland	616		
		Left Alliance	287		
	Other	Movement Now	94		
		Some other party	134		
		I would not vote	423		
EAI-12				4.91	0.86
SJS				3.36	0.68
VSA-3				3.21	0.76
Climate actions	I don’t know		67		
	I do not consider the environmental impact of my actions		150		
	I rarely consider the environmental impact of my actions		235		
	I sometimes consider the environmental impact of my actions		1254		
	I often consider the environmental impact of my actions		1644		
	I always consider the environmental impact of my actions		169		
Self-efficacy	Very difficult		42		
	Difficult		300		
	Not difficult nor easy		1619		
	Easy		1179		
	Very easy		197		
Climate concern	Not at all worried		239		
	Not too worried		606		
	Somewhat worried		1659		
	Very worried		799		
	Extremely worried		216		
CC knowledge	I do not know what climate change is		44		
	I have poor knowledge about climate change		114		
	I have mediocre knowledge about climate change		1062		
	I have good knowledge about climate change		1498		
	I have very good knowledge about climate change		801		
A Knowledge	I do not know what I can do to mitigate climate change		52		
	I know poorly what I can do to mitigate climate change		117		
	I roughly know what I can do to mitigate climate change		1156		
	I know fairly well what I can do to mitigate climate change		1413		
	I know very well what I can do to mitigate climate change		613		
	I do not know		168		
FinCA	Completely disagree		1160		
	Mostly disagree		917		
	Neither agree or disagree		596		
	Mostly agree		520		
	Completely agree		275		
	I do not know		51		
InCA	Completely disagree		208		
	Mostly disagree		368		

	Neither agree or disagree	653		
	Mostly agree	1377		
	Completely agree	849		
	I do not know	64		
Experience of climate change			2.81	1.09

Note: FinCA = There is no point in taking any actions to mitigate climate change in Finland, because most of the emissions are produced by other countries, InCA = The actions of individuals make a difference in mitigating climate change.

In addition to the variables presented in table S1.1, three sum variables (i.e. EAI-12, SJS, VSA-3) were used as explanatory variables. The original of the Environmental Attitudes Inventory (EAI) created by Milfont and Duckitt (2010) was shortened to a12 item measure. For the original EAI we selected one item from each of the original 12 EAI-scales. The items were selected mostly based on the developers´ shortened 24- item version. However, some exceptions were made to ensure that the items were appropriate to the Finnish context. Seven of the items (items from scales 1, 2, 3, 6, 8, 11, 12) addressed perseveration of nature and five of them utilization. Statements included in the EAI-12 measure are listed in table S1.2. Respondents indicated their agreement or disagreement with the statement on a seven-point scale (*Completely disagree, Disagree, Somewhat disagree, Neither disagree nor agree, Somewhat agree, Agree, Completely agree*). A unidimensional EAI-12 score was created by reversing the utilization items and then aggregating all of the items. The preservation factor of EAI-12 had Cronbach Alpha of .70 and utilization factor Alpha of .70 in our data signalling reasonable reliability. Distribution of the EAI-12 score is presented in figure S1.1

Table S1.2. EAI-12 statements in English and Finnish

English	Finnish
I think spending time in nature is boring. (R)	Minusta ajan viettäminen luonnossa on tylsää.
Controls should be placed on industry to protect the environment from pollution, even if it means things will cost more	Teollisuuden päästöjä tulisi rajoittaa säädöksillä, vaikka se tarkoittaisi tavaroiden hinnan nousua.
I would NOT get involved in an environmentalist organization. (R)	Minä EN osallistuisi ympäristöjärjestön toimintaan.
Conservation is important even if it lowers peoples' standard of living. (R, U)	Ympäristönsuojelu on tärkeää, vaikka se laskisi ihmisten elintasoa.
Modern science will solve our environmental problems. (U)	Tiede ja tekniikka tulevat ratkaisemaan ympäristöongelmat.
Humans are severely abusing the environment.	Ihmiset kohtelevat luontoa erittäin huonosti.
When nature is uncomfortable and inconvenient for humans we have every right to change and remake it to suit ourselves. (U)	Jos luonnontilainen ympäristö on ihmisille hankala paikka, heillä on täysi oikeus muokata se mieleisekseen.
I am NOT the kind of person who makes efforts to conserve natural resources.	Minä EN OLE sellainen ihminen, joka näkee vaivaa luonnonvarojen suojelemiseksi.
Humans were meant to rule over the rest of nature. (U)	Ihmiset on tarkoitettu hallitsemaan muuta luontoa.
Protecting peoples' jobs is more important than protecting the environment. (U)	Työpaikkojen suojeleminen on tärkeämpää kuin ympäristönsuojelu.
The idea that nature is valuable for its own sake is naïve and wrong. (R)	Ajatus siitä, että luonto olisi arvokas sen itsensä vuoksi on naiivi ja väärä.
We should strive for the goal of ''zero population growth''	Meidän tulisi pyrkiä tilanteeseen, jossa väestönkasvu pysähtyisi, eli ihmisten määrä maapallolla ei enää lisääntyisi.

Note: R: reversed scale, U: Utilization item

Three statements from the original VSA-scale by Bizumic and Duckitt (2018). were selected based on their suitability to Finnish culture. The statements are listed in table S1.3. Respondents indicated their agreement or disagreement with the statement on a five-point scale (*Strongly disagree, Somewhat disagree, Neither disagree nor agree, Fairly agree, Strongly agree*). A unidimensional VSA-3 score was created by aggregating the three items. The VSA-3 score had Cronbach Alpha of .60, signalling reasonable reliability. Distribution of aggregated VSA-3 score is presented in figure S1.1.

Table S1.3. VSA-3 statements in English and Finnish

English	Finnish
It’s great that many young people today are prepared to defy authority (R)	On hienoa, että moni nuori on nykyisin valmis vastustamaan auktoriteetteja.
What our country needs most is discipline, with everyone following our leaders in unity	Maamme tarvitsee ennen kaikkea kuria ja kaikkien tulisi yksimielisesti seurata päättäjiämme.
The facts on crime and the recent public disorders show we have to crack down harder on troublemakers, if we are going preserve law and order	Rikollisuuteen ja viimeaikaisiin levottomuuksiin liittyvät faktat osoittavat, että ongelmia aiheuttavat ihmiset on laitettava vahvemmin kuriin, jos aiomme ylläpitää lakia ja järjestystä.

Note: R: reversed scale

The eight modified statements form Kay and Jost’s (2003) System Justification Scale (SJS) that were used to measure system justification are listed in table S1.4. Respondents indicated their agreement or disagreement with the statement on a five-point scale (*Strongly disagree, Somewhat disagree, Neither disagree nor agree, Fairly agree, Strongly agree*). A unidimentional SJS consturct was created by aggregating the eight items. The SJS construct had Cronbach Alpha of .85, signalling good reliability. Distribution of aggregated SJS score is presented in figure S1.1.

Table S1.4. SJS statements in English and Finnish

English	Finnish
In general, Finnish society is fair and just.	Yleisesti ottaen suomalainen yhteiskunta on oikeudenmukainen ja reilu.
In general, the Finnish political system works as it should.	Yleisesti ottaen Suomen poliittinen järjestelmä toimii kuten sen pitääkin.
Finnish society needs to be radically restructured.	Suomalainen yhteiskunta tulisi järjestää perusteellisesti uudella tavalla.
Finland is the best country in the world to live in.	Suomi on maailman paras maa asua.
In Finland, most policies serve the greater good.	Suurin osa Suomen käytännöistä ja säädöksistä palvelevat yleistä hyvää.
In Finland, everyone has a fair shot at wealth and happiness	Suomessa jokaisella on hyvät mahdollisuudet vaurauteen ja hyvinvointiin.
Finnish society is getting worse every year.	Suomalainen yhteiskunta menee vuosi vuodelta huonommaksi.
Finnish society is set up so that people usually get what they deserve.	Suomalainen yhteiskunta on rakennettu siten, että ihmiset saavat yleensä ansionsa mukaan.

Figure S1.1. Distributions of aggregated EAI-12, VSA-3 and SJS scores

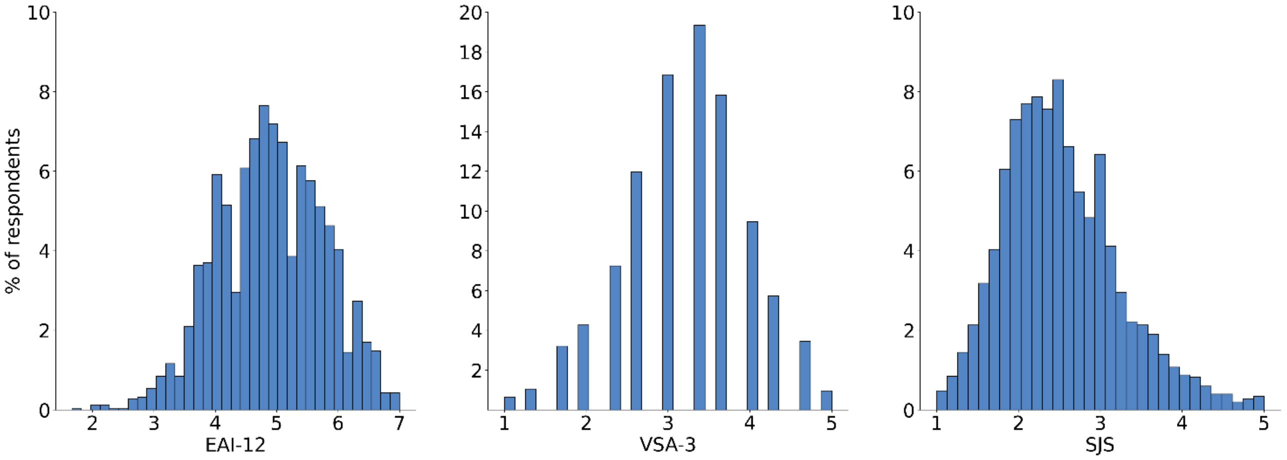

Calculation of the carbon footprint variables used as explanatory variables is described in S2 Appendix. The distributions of the variables obtained from the calculations are shown in figure S1.2.

Figure S1.2. Distrubutions of total carbon footprint per consumption unit and carbon footprint by the sectors

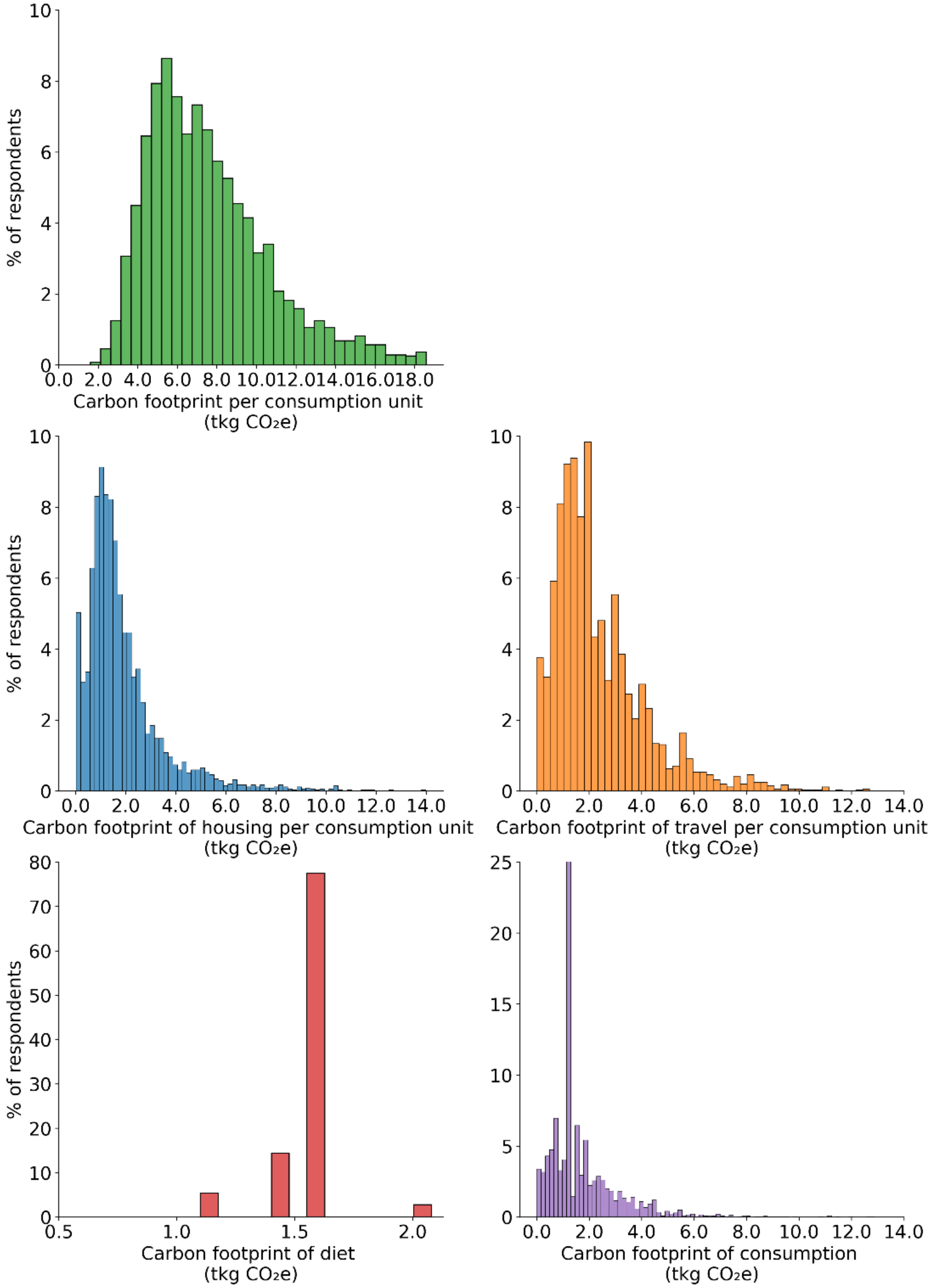

The results from ANOVAs with carbon footprint as dependent variable and gender, urbanicity of residential area and political positioning as independent variables are presented in tables S1.5-S1.7.

Table S1.5. Results from ANOVA with carbon footprint as dependent variable and gender as independent variable

Measure	Female		Male		F	p	η2
	M	SD	M	SD			
CF	7.12	2.89	8.12	3.18	95.81	<.001	0.03

Note: CF: Carbon footprint

Table S1.6. Results from ANOVA with carbon footprint as dependent variable and urbanicity as independent variable

Measure	Urban		Non-urban		F	p	η2
	M	SD	M	SD			
CF	7.39	3.01	7.96	3.14	27.04	<.001	0.01

Note: CF: Carbon footprint

Table S1.7. Results from ANOVA with carbon footprint as dependent variable and political positioning as independent variable

Measure	Right		Left		Other		F	p	η2
	M	SD	M	SD	M	SD			
CF	8.06	3.07	7.18	2.94	7.06	3.10	41.01	< .001	0.01

Note: CF: Carbon footprint

Determinants of carbon footprint

Impact of different factors to the size of total carbon footprint was studied using a hierarchical multivariate linear model. Independent variables were added to the model in two blocks. The first block included socio-demographic factors, i.e., gender, age, household income and urbanicity of the respondent's residential area. The second block included political positioning, climate concern, environmental attitude inventory (EAI-12) [47], System justification scale (SJS) [48] and Very Short Authoritarianism Scale (VSA-3) [49]. Both models explained variation in the size of the carbon footprint better than an empty model; model 1: $F(5,3311) = 125.09$, $p = <.001$, adjusted $R^2 = 0.16$, model 2: $F(6,3305) = 62.95$, $p = < .001$, adjusted $R^2 = 0.17$. The explanatory power of the second model was significantly higher than the first model´s, $F_{change}=9.55$, $p = < .001$. Results from the model are presented in table S1.8

Table S1.8. Results of the hierarchical multiple linear regression model with carbon footprint as dependent variable

Model		B	SE	Stand. B	[LL, LU]	p	ΔR²	VIF
1	Intercept	6.165	0.289	-	[5.421, 6.908]	< .001*	-	
	Age	0.007	0.003	.038	[-0.001, 0.014]	.018	.001	1.02
	Gender: Women[1]	-0.628	0.100	-.102	[-0.887, -0.370]	< .001*	.010	1.04
	Household income	0.543	0.028	.344	[0.472, 0.614]	< .001*	.099	1.2
	Level of education	0.078	0.055	.024	[-0.065, 0.220]	.161	< .001	1.19
	Urbanicity: Urban[2]	-0.679	0.107	-.103	[-0.954, -0.404]	< .001*	.010	1.03
2	Intercept	7.489	0.507	.000	[6.183, 8.794]	< .001*	.	.
	Age	0.008	0.003	.042	[0.000, 0.015]	.012	.	1.09
	Gender: Male[1]	-0.468	0.102	-.076	[-0.732, -0.204]	< .001*	.	1.1
	Household income	0.517	0.028	.328	[0.445, 0.588]	< .001*	.	1.23
	Level of education	0.131	0.057	.041	[-0.017, 0.278]	.022	.	1.29
	Urbanicity: Urban[2]	-0.630	0.107	-	[-0.905, -0.356]	< .001*	.	1.04
	Climate concern	-0.018	0.066	-.006	[-0.188, 0.152]	.784	< .001	1.7
	EAI-12	-0.363	0.075	-.102	[-0.557, -0.168]	< .001*	.006	1.8
	Politics: Left[3]	-0.169	0.121	-.026	[-0.482, 0.143]	.163	< .001	1.43
	Politics: Other[3]	-0.320	0.141	-.041	[-0.684, 0.044]	.024	.001	1.28
	SJS	0.042	0.078	.009	[-0.160, 0.243]	.593	< .001	1.21
	VSA-3	0.078	0.069	.019	[-0.101, 0.257]	.263	< .001	1.19

Note: 1: compared to men. 2 compared non-urban, 3: compared to right-wing, * significant at p-value < 0.01, ** variable with highest ΔR^2

Similar hierarchical linear regression models were conducted to analyse the determinants of the carbon footprint of housing, travel and other consumption of goods and services. The hierarchical model explaining the variation in the size of carbon footprint of housing per consumption unit explained the variation better than an empty model: model 1: F(5,3311) = 54.96, p = <.001, adjusted R^2 = 0.08, model 2: F(6,3305) = 26.35, p = <.001, adjusted R^2 = 0.08. Adding the second block of independent variables to the model increased the explanatory power of the model, F_{change}= 2.40, p = 0.07. The results are presented in table S1.9.

Table S1.9. Results of the explorative multiple linear regression with carbon footprint of housing as dependent variable

Model		B	SE	Standardised B	[LL, LU]	p	ΔR^2
1	Intercept	0.880	0.157	-	[0.476, 1.284]	< .001*	-
	Age	0.022	0.002	.235	[0.018, 0.026]	< .001*	.002
	Gender: Women[1]	-0.009	0.054	-.003	[-0.150, 0.131]	.866	.010
	Household income	0.063	0.015	.077	[0.024, 0.101]	< .001*	.100
	Level of education	0.034	0.030	.020	[-0.044, 0.111]	.263	< .001
	Urbanicity: Urban[2]	-0.322	0.058	-.094	[-0.472, -0.173]	< .001*	.003
2	Intercept	0.989	0.277	.000	[0.275, 1.703]	< .001*	
	Age	0.022	0.002	.237	[0.018, 0.027]	< .001*	
	Gender: Male[1]	0.031	0.056	.010	[-0.113, 0.176]	.576	
	Household income	0.058	0.015	.071	[0.019, 0.097]	< .001*	
	Level of education	0.058	0.031	.035	[-0.023, 0.139]	.065	
	Urbanicity: Urban[2]	-0.304	0.058	-.089	[-0.454, -0.154]	< .001*	
	Climate concern	-0.048	0.036	-.029	[-0.141, 0.045]	.181	< .001
	EAI-12	-0.054	0.041	-.029	[-0.161, 0.052]	.189	.006
	Politics: Left[3]	-0.015	0.066	-.005	[-0.186, 0.156]	.816	.001
	Politics: Other[3]	0.035	0.077	.009	[-0.164, 0.234]	.647	.001
	SJS	0.008	0.043	.003	[-0.103, 0.118]	.860	< .001
	VSA-3	0.052	0.038	.025	[-0.046, 0.150]	.171	< .001

Note: 1: compared to men. 2 compared non-urban, 3: compared to right-wing, * significant at p-value < 0.01, ** variable with highest ΔR^2

The hierarchical model explained the variation in the size of the carbon footprint of travel better than the empty model: model 1: F(5,3311) = 48.89, p = <.001, adjusted R^2 = 0.07, model 2: F(6, 3305) = 26.87, p = <.001, adjusted R^2 = 0.08. Adding the second block of independent variables to the model significantly increased the explanatory power of the model significantly, F_{change}= 8.01, p = < .001. The results are presented in table S1.10.

Table S1.10. Results of the hierarchical multiple linear regression model with carbon footprint of travel as dependent variable

Model		B	SE	Standardised B	[LL, LU]	p	ΔR^2
1	Intercept	3.349	0.176	-	[2.895, 3.803]	< .001*	-
	Age	-0.016	0.002	-.148	[-0.020, -0.011]	< .001*	.022
	Gender: Women[1]	-0.351	0.061	-.098	[-0.509, -0.193]	< .001*	.009
	Household income	0.167	0.017	.182	[0.123, 0.210]	< .001*	.028
	Level of education	-0.025	0.034	-.013	[-0.112, 0.063]	.467	< .001
	Urbanicity: Urban[2]	-0.357	0.065	-.093	[-0.525, -0.188]	< .001*	.008
2	Intercept	3.967	0.310	.000	[3.169, 4.766]	< .001*	
	Age	-0.015	0.002	-.144	[-0.020, -0.010]	< .001*	
	Gender: Male[1]	-0.263	0.063	-.073	[-0.424, -0.102]	< .001*	
	Household income	0.156	0.017	.171	[0.113, 0.200]	< .001*	
	Level of education	0.016	0.035	.008	[-0.075, 0.106]	.658	
	Urbanicity: Urban[2]	-0.323	0.065	-.084	[-0.491, -0.155]	< .001*	
	Climate concern	-0.064	0.040	-.035	[-0.168, 0.040]	.111	.001
	EAI-12	-0.130	0.046	-.063	[-0.249, -0.011]	.005	.002
	Politics: Left[3]	-0.050	0.074	-.013	[-0.241, 0.141]	.500	< .001
	Politics: Other[3]	-0.208	0.086	-.045	[-0.430, 0.015]	.016	.002
	SJS	-0.065	0.048	-.025	[-0.188, 0.059]	.177	.001
	VSA-3	0.094	0.042	.040	[-0.015, 0.204]	.026	.001

Note: 1: compared to men. 2 compared non-urban, 3: compared to right-wing, * significant at p-value < 0.01, ** variable with highest ΔR^2

The hierarchical model explained variation in the size of carbon footprint of consumption better than an empty model: model 1: $F(5,3311) = 186.03$, p = <.001, adjusted $R^2 = 0.22$, model 2: $F(6,3305) = 89.68$, p = <0.01, adjusted $R^2 = 0.23$. Adding the second block of independent variables significantly increased the explanatory power of the model, F_{change}= 7.56, p = < .001. The results are presented in table S1.11.

Table S1.11. Results of the hierarchical multiple linear regression model with carbon footprint of consumption as dependent variable

Model		B	SE	Standardised B	[LL, LU]	p	ΔR^2
1	Intercept	0.322	0.126	-	[-0.002, 0.645]	.010	-
	Age	0.000	0.001	.006	[-0.003, 0.004]	.719	< .001
	Gender: Women[1]	-0.223	0.044	-.08	[-0.335, -0.110]	< .001*	.006
	Household income	0.301	0.012	.423	[0.271, 0.332]	< .001*	.149
	Level of education	0.084	0.024	.058	[0.022, 0.146]	< .001*	.003
	Urbanicity: Urban[2]	0.015	0.046	.005	[-0.105, 0.135]	.743	< .001
2	Intercept	0.862	0.221	.000	[0.292, 1.431]	< .001*	
	Age	0.000	0.001	.005	[-0.003, 0.004]	.763	
	Gender: Women[1]	-0.209	0.045	-.075	[-0.324, -0.093]	< .001*	
	Household income	0.293	0.012	.411	[0.262, 0.324]	< .001*	.004
	Level of education	0.065	0.025	.045	[0.000, 0.129]	.010	.005
	Urbanicity: Urban[2]	0.005	0.047	-	[-0.115, 0.125]	.916	.001
	Climate concern	0.114	0.029	.079	[0.040, 0.188]	< .001*	.001
	EAI-12	-0.153	0.033	-.095	[-0.237, -0.068]	< .001*	.001
	Politics: Left[3]	-0.095	0.053	-.033	[-0.231, 0.041]	.073	.002
	Politics: Other[3]	-0.152	0.062	-.043	[-0.311, 0.007]	.014	
	SJS	0.086	0.034	.042	[-0.002, 0.174]	.012	
	VSA-3	-0.084	0.030	-.046	[-0.163, -0.006]	.005	

Note: 1: compared to men. 2 compared non-urban, 3: compared to right-wing, * significant at p-value < 0.01, ** variable with highest ΔR^2

The determinants of the carbon footprint of diet were explored using logistic regression models, similar to the linear regression models used in other analyses, albeit with reduced variable categories (see Table 6). Average to high meat diet was used as the reference group. The results from the initial model is presented in Supplementary material that included only sociodemographic factors demonstrated a significant improvement in fit compared to the intercept-only model ($\chi^2 = 272.74$, $p < .001$, Nagelkerke R-squared = 0.11). The second model also showed a significant improvement over the intercept-only model ($\chi^2 = 496.23$, $p < .001$, Nagelkerke R-squared = 0.20). The results are presented in tables S1.12. and S1.13.

Table S1.12. Multinomial logistic regression model with carbon footprint of diet as dependent variable and sociodemographic factors as independent variables. Reference category is high meat diet. Risk is presented as Exp(B) odd ratios.

		Vegan or pesco-vegetarian			Low meat		
Variable		p	OR	99 % CI	p	OR	99 % CI
Age	≥ 65 years	< .001*	0.33	[0.17, 0.63]	.010	1.69	[1.00, 2.86]
	50–64 years	< .001*	0.29	[0.14, 0.60]	.407	1.2	[0.69, 2.08]
	40–49 years	< .001*	0.38	[0.19, 0.79]	.043	0.61	[0.33, 1.14]
	30–39 years	.463	0.84	[0.45, 1.56]	.871	0.96	[0.53, 1.76]
	≤ 29 years	.	.	.	.	.	.
Gender	Women	< .001*	3.6	[2.10, 6.18]	< .001*	1.5	[1.14, 1.98]
	Men	.	.	.	.	.	.
Household income	≥ 7 500 €	.144	0.46	[0.11, 1.82]	.013	0.45	[0.19, 1.03]
	3 500 - 7 499 €	< .001*	0.47	[0.28, 0.77]	< .001*	0.53	[0.38, 0.72]
	2 500- 3 499 €	.003	0.49	[0.27, 0.91]	< .001*	0.56	[0.39, 0.80]
	≤ 2 500 €	.	.	.	.	.	.
Education	2. level or lower	< .001*	0.56	[0.36, 0.87]	< .001*	0.6	[0.45, 0.79]
	3. level or higher	.	.	.	.	.	.
Urbanicity	Non-urban	.002	0.51	[0.29, 0.88]	.855	1.02	[0.77, 1.36]
	Urban	.	.	.	.	.	.

Note: CC = climate change, CA = climate action, FiCA = Climate actions in Finland, * significant at p-value < 0.01, ** variable with highest significant OR.

Table S1.13. Multinominal logistic regression model with carbon footprint of diet as dependent variable. Reference category is high meat diet. Risk is presented as Exp(B) odd ratios.

		Vegan or pesco-vegetarian			Low meat		
Variable		p	OR	99 % CI	p	OR	99 % CI
Age	≥ 65 years	< .001*	0.36	[0.18, 0.73]	.010	1.72	[1.00, 2.95]
	50–64 years	< .001*	0.28	[0.13, 0.61]	.423	1.19	[0.68, 2.09]
	40–49 years	.001	0.37	[0.17, 0.81]	.035	0.59	[0.31, 1.12]
	30–39 years	.207	0.72	[0.36, 1.42]	.799	0.94	[0.51, 1.73]
	≤ 29 years	.	.	.	.	.	.
Gender	Women	< .001*	2.75	[1.56, 4.86]	.020	1.29	[0.97, 1.71]
	Men	.	.	.	.	.	.
Household income	≥ 7 500 €	.530	0.7	[0.16, 3.08]	.045	0.52	[0.22, 1.21]
	3 500 - 7 499 €	.008	0.58	[0.34, 0.99]	< .001*	0.55	[0.40, 0.76]
	2 500- 3 499 €	.023	0.56	[0.30, 1.08]	< .001*	0.57	[0.39, 0.82]
	≤ 2 500 €	.	.	.	.	.	.
Education	2. level or lower	.172	0.77	[0.47, 1.26]	< .001*	0.63	[0.47, 0.85]
	3. level or higher	.	.	.	.	.	.
Urbanicity	Non-urban	.024	0.6	[0.33, 1.07]	.411	1.1	[0.82, 1.47]
	Urban	.	.	.	.	.	.
CC concern	Not at all worried	.011	0.14	[0.02, 1.03]	.014	0.43	[0.18, 1.04]
	Not too worried	.010	0.38	[0.15, 1.00]	.005	0.47	[0.24, 0.94]
	Somewhat worried	< .001*	0.26	[0.13, 0.52]	.090	0.69	[0.40, 1.21]
	Very worried	.002	0.47	[0.25, 0.88]	.521	0.87	[0.50, 1.51]
	Extremely worried	.	.	.	.	.	.
EAI-12	Increase by 1 point	< .001*	1.93	[1.40, 2.68]	.027	1.18	[0.97, 1.43]
Politics	Left-wing	.051	1.55	[0.87, 2.75]	.236	1.16	[0.84, 1.60]
	Other	.507	1.21	[0.57, 2.57]	.121	1.27	[0.86, 1.88]
	Right-wing	.	.	.	.	.	.
VSA-3	Increase by 1 point	.002	0.71	[0.53, 0.94]	.011	0.84	[0.71, 1.00]
SJS	Increase by 1 point	.112	0.82	[0.59, 1.14]	.015	0.83	[0.68, 1.01]

Note: CC = climate change, CA = climate action, FiCA = Climate actions in Finland, * significant at p-value < 0.01, ** variable with highest significant OR.

S2 Supplementary material

This supplementary material provides description on the procedure to estimate carbon footprints of the survey respondents in the study.

1 Shared resources

Estimating carbon footprints per person calls for allocating emissions from total consumption of the household among household members. Table S2.1 distinguishes when footprints have been shared among household members and when considered as personal consumption. To share footprint among household members, we have used OECD consumption unit (1) to do this. According to the definition, the first adult aged 18 and over equal 1; subsequent adults aged 18 and over equal 0.7 and each person aged under 18 equals 0.5.

Table S2.1. Carbon footprint allocation principles for each consumption domain.

Carbon footprint	Personal	Consumption units
Energy consumption at home		x
Second home carbon footprint		x
Passenger car travel		x
Boat ownership and use		x
Travel with all other modes of transport	x	
Diet	x	
Expenditure in euros for other goods and services	x	

We are aware of real-life differences and other possible allocation approaches of carbon footprints across consumption domains. Resources may or may not be shared equally among household members. Also, sometimes resources such as boats, second homes or cars can be shared with people other than household members. Also, expenditure patterns may follow shared responsibilities among household members and not fully reflect personal consumption preferences. Nevertheless, it is justified to allocate the carbon footprint of resources that are or can be shared among household members.

Next, we present calculation principles and emission intensities including their data sources for each consumption domain included in this research.

2 Energy use at home

Heating energy consumption is considered as a key component of housing carbon footprint globally (2). This is highlighted in Finland, a country located in Northern Europe. To estimate the heating energy consumption properly, respondents were asked to indicate several characteristics of their homes. The questions included the size of their home, type of housing, heating energy sources, type of electricity contract, and the age of the building. These characteristics combined with number of people in household and their place of residence were used to estimate energy consumption for space- and water heating, and electricity use for other purposes at home. Table S2.2 lists assumptions used in estimation of household energy consumption.

Table S2.2. Energy consumption estimates.

Definition	Value and unit	Sources
Average space heating energy consumption	One family houses: 169 kWh per m^2 per year.<br><br>Row houses: 119 kWh per m^2 per year.<br><br>Apartments: 123 kWh per m^2 per year.	Statistics Finland StatFin database: Heating of residential buildings by building type in 2020 and number of buildings by regions and by intended use in 2020. Consumption estimates were adjusted to represent values for living area respondents provided.
Adjustment of space heating estimate by age of the building	Building year 2000-2010: no correction.<br><br>Build before 2000, multiplier to adjust energy consumption: one family houses 1.26; row houses 1.31; apartments 1.35.<br><br>Build 2010-2020, multiplier: one family houses and row houses 0.66, apartments 0.65.<br><br>Build after 2020, multiplier: one family houses 0.65, row houses 0.64, apartments 0.57.	Estimates were adjusted based on SYKE's calculation models taking into account changes in building codes affecting energy efficiency of building stock.
Adjustment of space heating energy based on residential location	Space heating energy estimates were adjusted based on heating degree data. Postal codes of respondents were used to determine municipality of the resident corresponding heating degree data.	(3)
Estimated energy consumption for water heating	One family and row houses: 42 kWh per m^2 per year.<br><br>Apartments: 49 kWh per m^2 per year.	Statistics Finland StatFin database: Heating of residential buildings by building type in 2020 and Energy source for space heating by type of building (4). Consumption estimates

		were adjusted according to living area respondents provided.
Electricity consumption estimate for appliances (i.e., excluding consumption for water and space heating).	Consumption estimate is presented in kWh per year per household depending on housing type:<br><br>One family houses: 4600 + (number of persons in household – 1) *900<br>Row houses: 2600 + (number of persons in household – 1) *700<br>Apartments: 1400 + (number of persons in household – 1) *500	
Ground source heating pump coefficient of performance (COP)	3	(5) (5)

Table S2.3. presents emission intensities use in calculating carbon footprint from housing energy consumption.

Table S2.3. Carbon intensity of household energy consumption.

**Definition**	**Emission intensity and unit**	**Sources**
Electricity, green or carbon neutral electricity subscription	0.011 kg CO_2e per kWh	Life-cycle emissions of wind power (6)
Electricity, average from Finnish grid (benefit allocation method, combustion and procurement of fuels)	0.153 kg CO_2e per kWh	Life-cycle emissions intensities for year 2020 (7).
District heat, Finnish average (benefit allocation method, combustion and procurement of fuels)	0.147 kg CO_2e per kWh	
Oil (fossil fuels, decentralized heating)	0.306 kg CO_2e per kWh	
Wood (biofuels, decentralized heating)	0.027 kg CO_2e per kWh	
Carbon footprint of second home heating (based on data from year 2020)	333 kg CO_2e per year	Own calculation based on Statistics Finland, StatFin database: Heating of residential buildings by building type (8) and number of buildings by regions and by intended use (8). Emission intensities used in calculation as presented above.

3 Travel

Respondents were asked to provide information on number and type of cars in household. Options for type of cars: gasoline/diesel, liquefied natural or biogas, self-charging hybrid, plug-in hybrid, electric. Length of trips and driving kilometres were asked in ranges, which were converted to total annual kilometres.

Emission intensities of transport modes and boat are listed in Table S2.4. Car manufacturing emissions are applied in calculations as follows: Car owning households are allocated manufacturing emissions for one year. The total lifespan of a car is estimated to be 22 years as according to Statistics Finland (9), that is the average age of removing passenger car from the vehicle registry in Finland in year 2021. Respondents who do not own a car but have used one are allocated manufacturing emissions on a kilometre basis. I.e., the yearly manufacturing emissions are divided by 14400 km.

Table S2.4. Carbon intensity estimates for travel.

**Definition**	**Emission intensity and unit**	**Sources**
Car driving, internal combustion engine (gasoline)	0.213 kg CO_2e per vehicle km	(10) and (7) for electricity emissions during use phase. Electricity emissions average from Finnish grid as presented in housing section.
Car driving, self-charging hybrid	0.165 kg CO_2e per vehicle km	
Car driving, plug-in hybrid	0.117 kg CO_2e per vehicle km	
Car driving, electric car	0.026 kg CO_2e per vehicle km	
Car driving, liquified natural/biogas	0.116 kg CO_2e per vehicle km	
Car manufacturing, internal combustion engine (gasoline or liquified natural/biogas)	287 kg CO_2e per vehicle per year	(10)
Car manufacturing, self-charging or plug-in hybrid	311 kg CO_2e per vehicle per year	
Car manufacturing, electric car	353 kg CO_2e per vehicle per year	
Air travel (Including: direct fuel emissions during flight, fuel manufacturing, aircraft manufacturing. Excluding: Radiative Forcing Index)	0.116 kg CO_2e per passenger km	Direct SCOPE 1 emissions (11), fuel life-cycle emissions (12), aircraft manufacturing (13).
Bus travel (weighted average from city and long-haul traffic in Finland)	0.069 kg CO_2e per passenger km	Direct emissions from fuel combustion (14), fuel life cycle emissions (15), vehicle manufacturing (16)
Train travel (operating power, mostly electricity emissions from renewable sources and train manufacturing)	0.007 kg CO_2e per passenger km	Direct emissions from electricity and fuel (17), renewable electricity indirect emissions estimated based on (6), train manufacturing (13).
Ferry Helsinki-Tallinn one way	10 kg CO_2e per trip per passenger	

Ferry Helsinki-Stockholm one way	31 kg CO_2e per trip per passenger	Direct CO_2 emissions from fuel (18), fuel life cycle emissions (19), ship manufacturing (20).
Rowing boat	48 kg CO_2e per boat per year	Own calculations based on data from (21). Estimated service life of boats 30 years.
Motorboat (including estimated fuel use)	572 kg CO_2e per boat per year	
Sailing boat	436 kg CO_2e per boat per year	

4 Diet

Responses on food consumption were translated to indicate the type of diet. Saarinen et al. (22) studied greenhouse gas emissions of different diets based on Finnish food consumption patterns. The study distinguishes vegan, pesco-vegetarian, low-meat, and average Finnish diet. Based on their findings, we estimated diet with high meat consumption. Diet emissions include the life cycle emissions of food consumed but excludes emissions form energy consumption of cooking at home as that is included in home energy consumption. The estimated yearly greenhouse gas emissions from food consumption (excluding land use emissions) are listed in Table S2.5.

Table S2.5. Carbon footprint of diet options.

Diet	Carbon footprint
Vegan	1100 kg CO_2e per person per year
Pesco-vegetarian	1130 kg CO_2e per person per year
Low meat	1420 kg CO_2e per person per year
Average	1610 kg CO_2e per person per year
High meat consumption	2080 kg CO_2e per person per year

5 Consumption of other goods and services

Respondents were asked to choose from a range on their monthly expenditure on other goods and services. Ranges were converted into yearly expenditure.

Emission intensity estimations for expenditure are based on (23). Yearly expenditure patterns of respondents were first converted to value in year 2015 (24) as the emission intensities are indicated in 2015 values. The emission intensity data is from year 2019, the latest data available at the time of writing. Intensities data is based on ENVIMAT, environmentally extended input-output model of the Finnish economy (23,25). Greenhouse gas intensities of household consumption expenditure is available for 65 commodities in total

classified according to international Coicop (26) classification. The emission intensities used are listed in Table S2.6.

Table S2.6. Carbon intensity estimates for consumption of other goods and services.

**Expenditure category in survey**	**Emission intensity**
Furnishings, household equipment and home appliances	0.4 kg CO_2e per euro
Clothing and footwear	0.3 kg CO_2e per euro
Electronic appliances such as computers and mobile phones	0.6 kg CO_2e per euro
Expenditure on other items	0.4 kg CO_2e per euro
Recreation and services	0.2 kg CO_2e per euro

The carbon footprint estimations were performed utilizing Python 3.9. The script, along with its corresponding parameters, is available in https://osf.io/g6x8f/.

References

1. Statistics Finland. Definition consumption unit (OECD) [Internet]. 2022 [cited 2022 Aug 17]. Available from: https://www.stat.fi/meta/kas/kulutusyks_en.html

2. Girod B, van Vuuren DP, Hertwich EG. Climate policy through changing consumption choices: Options and obstacles for reducing greenhouse gas emissions. Glob Environ Change. 2014 Mar 1;25:5–15.

3. Finnish meteorological institute. Heating degree days. Data for 1981-2010 [Internet]. [cited 2022 Aug 17]. Available from: https://en.ilmatieteenlaitos.fi/heating-degree-days

4. Statistics Finland. Energy sources for space heating by type of building [Internet]. 2021 [cited 2022 Aug 29]. Available from: https://pxhopea2.stat.fi/sahkoiset_julkaisut/energia2021/html/suom0006.htm

5. Motiva. Maalämpöpumppu [Internet]. [cited 2022 Aug 17]. Available from: https://www.motiva.fi/ratkaisut/uusiutuva_energia/lampopumput/lampopumpputeknologiat/maalampopumppu

6. Scarlat N, Prussi M, Padella M. Quantification of the carbon intensity of electricity produced and used in Europe. Appl Energy. 2022 Jan;305:117901.

7. Finnish Environment Institute and Ministry of the Environment Finland. Emissions database for construction. [cited 2022 Aug 17]. Emissions database for construction. Available from: https://co2data.fi/

8. Statistics Finland. StatFin, Heating of residential buildings by building type [Internet]. 2022 [cited 2022 Aug 17]. Available from: https://statfin.stat.fi/PxWeb/pxweb/en/StatFin/StatFin__asen/statfin_asen_pxt_11zr.px/

9. Statistics Finland. StatFin, Vehicles removed from the vehicle register [Internet]. 2022 [cited 2022 Aug 17]. Available from: https://statfin.stat.fi/PxWeb/pxweb/en/StatFin/StatFin__mkan/statfin_mkan_pxt_11tq.px/

10. The Finnish Climate Change Panel. Autokalkulaattori (car carbon footprint calculator). Available from: https://www.ilmastopaneeli.fi/autokalkulaattori/

11. Finnair. Vuosikertomus 2020 [Internet]. 2021 [cited 2022 Aug 17]. Available from: https://investors.finnair.com/~/media/Files/F/Finnair-IR/documents/fi/reports-and-presentation/2021/vuosikertomus-2020.pdf

12. Prussi M, Lee U, Wang M, Malina R, Valin H, Taheripour F, et al. CORSIA: The first internationally adopted approach to calculate life-cycle GHG emissions for aviation fuels. Renew Sustain Energy Rev. 2021 Oct;150:111398.

13. Chester MV, Horvath A. Environmental assessment of passenger transportation should include infrastructure and supply chains. Environ Res Lett. 2009 Apr;4(2):024008.

14. VTT. LIPASTO traffic emissions [Internet]. [cited 2022 Jul 11]. Available from: http://www.lipasto.vtt.fi/en/index.htm

15. European Commission. Study on Actual GHG Data Oil Gas Final Report [Internet]. 2015. Available from: https://ec.europa.eu/energy/sites/ener/files/documents/Study%20on%20Actual%20GHG%20Data%20Oil%20Gas%20Final%20Report.pdf

16. Nordelöf A, Romare M, Tivander J. Life cycle assessment of city buses powered by electricity, hydrogenated vegetable oil or diesel. Transp Res Part Transp Environ. 2019 Oct;75:211–22.

17. VR Group. Vastuullisuusraportti 2021 [Internet]. 2022 [cited 2022 Aug 17]. Available from: https://www.vrgroup.fi/fi/vuosiraportti-2021/?goto=/fi/vuosiraportti-2021/vuosiraportin-kuvaus/

18. Finnish Shipowners' Association. Merenkulun hiilidioksidipäästöt [Internet]. [cited 2022 Aug 17]. Available from: https://shipowners.fi/vastuullisuus/ymparisto/ilmastosuojelu-ja-ilmastonmuutos/merenkulun-hiilidioksidipaastot/

19. Comer B, Osipova L. Accounting for well-to-wake carbon dioxide equivalent emissions in maritime transportation climate policies [Internet]. International council on clean transportation; 2021. (Briefing). Available from: https://theicct.org/sites/default/files/publications/Well-to-wake-co2-mar2021-2.pdf

20. Simonsen M. CRUISE SHIP TOURISM - A LCA ANALYSIS [Internet]. Western Norway Research Institution; 2014. Available from: http://transport.vestforsk.no/Dokumentasjon/pdf/Skip/Cruise.pdf

21. Tonteri H, Auvinen H, Helin T, Johansson M. Ympäristömyötäisyyden kehittäminen venealalla [Internet]. VTT; 2010. Report No.: VTT-R-02928-10. Available from: https://publications.vtt.fi/julkaisut/muut/2010/VTT_R_02928_10.pdf

22. Saarinen M, Kaljonen M, Niemi J, Antikainen R, Hakala K, Hartkainen H, et al. Ruokavaliomuutoksen vaikutukset ja muutosta tukevat politiikkayhdistelmät. RuokaMinimi-hankkeen loppuraportti [Internet]. 2019 p. 160. (Valtioneuvoston selvitys- ja tutkimustoiminnan julkaisusarja). Report No.: 47. Available from: https://julkaisut.valtioneuvosto.fi/bitstream/handle/10024/161742/VNTEAS_47_Ruokavaliomuutoksen%20vaikutukset.pdf?sequence=1&isAllowed=y

23. Nissinen A, Savolainen H. Carbon footprint and raw material requirement of public procurement and household consumption in Finland. 2019 p. 70. (Reports of the Finnish Environment Institute). Report No.: 15en/2019.

24. Statistics Finland. StatFin, Consumer price index [Internet]. 2022 [cited 2022 Aug 17]. Available from: https://pxweb2.stat.fi/PxWeb/pxweb/en/StatFin/StatFin__khi/

25. Seppälä J, Mäenpää I, Koskela S, Mattila T, Nissinen A, Katajajuuri JM, et al. An assessment of greenhouse gas emissions and material flows caused by the Finnish economy using the ENVIMAT model. J Clean Prod. 2011 Nov;19(16):1833–41.

26. Statistics Finland. Coicop [Internet]. 2022 [cited 2022 Aug 19]. Available from: https://www.stat.fi/en/luokitukset/coicop/
